# (*E*)-2-Benzylidenecyclanones: Part XVIII Study the Possible Link between Glutathione Reactivity and Cancer Cell Cytotoxic Effects of Some Cyclic Chalcone Analogs A Comparison of the Reactivity of the Open-Chain and the Seven-Membered Homologs

**DOI:** 10.3390/ijms24108557

**Published:** 2023-05-10

**Authors:** Fatemeh Kenari, Szilárd Molnár, Igor D. Borges, Hamilton B. Napolitano, Pál Perjési

**Affiliations:** 1 Institute of Pharmaceutical Chemistry, University of Pécs, H-7624 Pécs, Hungary; kenari.fatemeh@pte.hu (F.K.); molnar.szilard@pte.hu (S.M.); 2 Research Institute for Viticulture and Oenology, University of Pécs, H-7634 Pécs, Hungary; 3 Grupo de Química Teórica e Estrutural de Anápolis, Universidade Estadual de Goiás, Anápolis 75070-290, GO, Brazilhamilton@ueg.br (H.B.N.)

**Keywords:** chalcone, glutathione, cysteine, thiols, Michael addition, diastereoselective addition

## Abstract

Non-enzymatic thiol addition into the α,β-unsaturated carbonyl system is associated with several biological effects. In vivo, the reactions can form small-molecule thiol (e.g., glutathione) or protein thiol adducts. The reaction of two synthetic (4′-methyl- and 4′-methoxy substituted) cyclic chalcone analogs with reduced glutathione (GSH) and *N*-acetylcysteine (NAC) was studied by (high-pressure liquid chromatography-ultraviolet spectroscopy) HPLC-UV method. The selected compounds displayed in vitro cancer cell cytotoxicity (IC_50_) of different orders of magnitude. The structure of the formed adducts was confirmed by (high-pressure liquid chromatography-mass spectrometry) HPLC-MS. The incubations were performed under three different pH conditions (pH 3.2/3.7, 6.3/6.8, and 8.0/7.4). The chalcones intrinsically reacted with both thiols under all incubation conditions. The initial rates and compositions of the final mixtures depended on the substitution and the pH. The frontier molecular orbitals and the Fukui function were carried out to investigate the effects on open-chain and seven-membered cyclic analogs. Furthermore, machine learning protocols were used to provide more insights into physicochemical properties and to support the different thiol-reactivity. HPLC analysis indicated diastereoselectivity of the reactions. The observed reactivities do not directly relate to the different in vitro cancer cell cytotoxicity of the compounds.

## 1. Introduction

Chalcones (**I**) are natural products, the biosynthetic precursors of flavonoids, a large family of plant phenolic secondary metabolites [1,2]. Because of the wide range of beneficial biological actions of the natural chalcones, several analogs have been synthesized and—among others—tested for their antioxidant, antimicrobial, antiprotozoal, antiulcer, antihistaminic, antidiabetic, anti-inflammatory, anticancer and neuroprotective activities [3,4,5,6]. The molecular mechanisms of the published biological/pharmacological effects can be associated with their (a) non-covalent interactions with biological macromolecules and (b) covalent modification of preferably the soft nucleophilic thiol function(s) of amino acids, peptides, and proteins [7,8,9].

**Figure 1:**
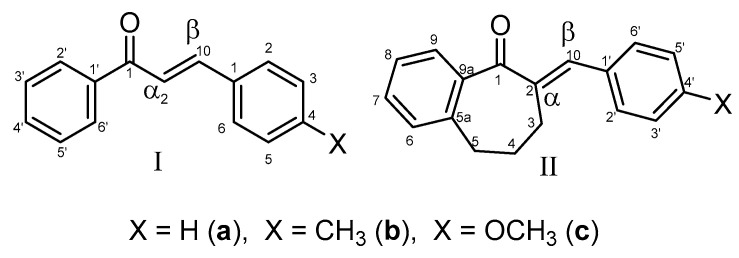
Structure and numbering of 4-X-chalcones (**I**) and (*E*)-2-(4′-X-phenylmethylene-1-benzosuberones (**II**).

The chalcone structure can be divided into three different structural units: the aromatic rings A and B and the propenone linker (Figure 1). Modifying any of them can tune the main feature of interactions of the synthetic chalcones towards the non-covalent or the covalent pathway. In our previous studies, we have investigated how the substitution of the B-ring and modifying the ring size (n = 5–7) of cyclic chalcone analogs can affect the cancer cell cytotoxic effect of more than one hundred twenty derivatives [10,11,12]. While comparing the average IC_50_ values of the series, the benzosuberone (**II**) analogs proved to be the most effective against P388, L1210, Molt 4/C8, and CEM cells, as well as a panel of human tumor cell lines. In particular, the (*E*)-2-(4-methoxyphenylmethylene)-1-benzosuberone (**II c**) had the most remarkable cytotoxicity, when all five screens were considered [10,11].

In consecutive publications, we have performed cell cycle analysis of Jurkat cells exposed to **IIc** and its methyl-substituted analogue **IIb**. It was demonstrated that equitoxic doses of the two cyclic chalcone analogs have different effects on the cell cycle progression of the investigated Jurkat cells. Compound **IIc** showed to cause an immediate G1 lift and G2/M arrest, followed by hypoploidity and aneuploidy. Such a remarkable effect of **IIb** on the G1 and G2 checkpoints could not be observed [13,14]. Thin layer chromatographic (TLC) and high-pressure liquid chromatographic (HPLC) analysis showed the compounds to react with reduced glutathione (GSH) under basic conditions [13,15]. However, the two compounds had different effects on the thiol status of the Jurkat cells [16]. 

Glutathione is ubiquitous in mammalian cells ranging in 1–10 mM concentrations [17]. Under physiological conditions, more than 98% of total glutathione occurs in reduced form [18,19]. The reduced glutathione (GSH)/oxidized glutathione (GSSG) (GSH/GSSG) redox system has a vital role in maintaining the (a) environment of the intracellular redox system, (b) the antioxidant defense system, and (c) the cellular signaling processes [20]. Furthermore, it is one of the endogenous substances involved in the metabolism of endogenous (e.g., estrogens, leukotrienes, prostaglandins) and exogenous compounds (e.g., drugs, non-energy-producing xenobiotics) [21].

Covalent bond formation of GSH with electrophilic species affects the half-cell reduction potential of the GSSG/2GSH redox system. The GSH/GSSG ratio is a critical mechanism for cell survival; in fact, it is known that it varies in association with proliferation, differentiation, and apoptosis [22,23]. In our earlier publication [24], we reported on the thiol reactivity of two open-chain chalcones (**Ib** and **Ic**) with different cancer cell cytotoxicities [25]. We could not find a direct correlation of the thiol reactivities and the previously published biological (cancer cell cytotoxic) effects of the two chalcones. Continuing the previous studies on the molecular mechanism of the cancer cell cytotoxic and cell cycle modulating effects of **IIb** and **IIc**, we report on a comparative HPLC study on their intrinsic reactivity towards GSH and N-acetylcysteine (NAC). Compound **IIc** showed IC_50_ values towards the most investigated cancer cell lines close to two magnitudes lower than the 4-methyl analog **IIb** (Table 1). Thus, the differences in the reactivities could reflect the differences in their previously published biological activities.

Similar to the previous publication [24], the reactions were studied under three conditions with different pH: (a) pH 8.0/7.4, (b) pH 6.3/6.8, and (c) pH 3.2/3.7. The first pH values indicate the pH of the aqueous solution of the thiols before starting the incubations; the second pH values indicate the virtual pH of the incubation mixtures, containing 75.5% *v/v* methanol (MeOH). The basic pH was selected to mimic the conditions of the GST-catalyzed reactions; the ionization of the GSH thiol-moiety is increased due to its interaction with the basic imidazole N-atom in the active site of the enzyme [26]. The pH 6.3 was selected to mimic the slightly acidic pH of cancerous cells [27], while a strongly acidic condition (pH 3.2) was chosen to compare how the thiol function of the compounds reacts in its protonated and ionized forms. GSH and NAC have reported pKa values of 8.83 and 9.52, respectively. Accordingly, the thiol function of both compounds exists exclusively in the protonated (neutral) form under the pH 3.2/3.7 conditions [28].

The thiol additions to enones are reported to be reversible, resulting in an equilibrium mixture’s formation. To qualitatively characterize the progress of the reactions, the composition of the incubation mixtures was analyzed at the 15, 45, 75, 105, 135, 165, 195, 225, 255, 285, and 315 min timepoints by HPLC-UV. Furthermore, density functional theory (DFT) calculations and machine learning (ML) protocols were used to analyze the stability and regioselectivity of chalcone analogs on a structural basis. In the analyses, methanethiol (**CH_3_SH**) and its deprotonated form (**CH_3_S^−^**) were used as model thiols.

## 2. Results

### 2.1. Reactions under Slightly Basic (pH 8.0/7.4) Conditions

**Figure 2:**
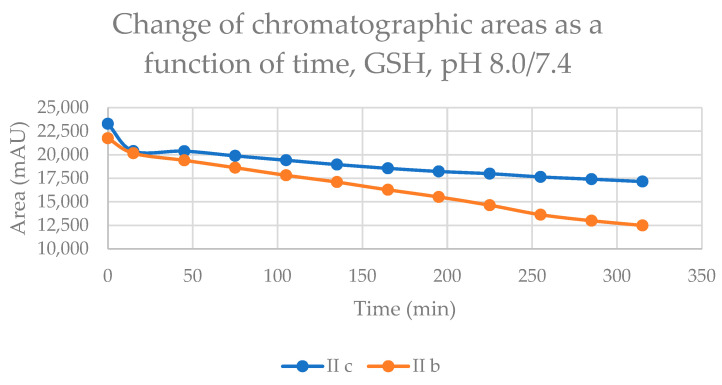
Change in the chromatographic peak area of chalcones **IIb** and **IIc** in the chalcone–GSH incubations at pH 8.0/7.4.

**Figure 3:**
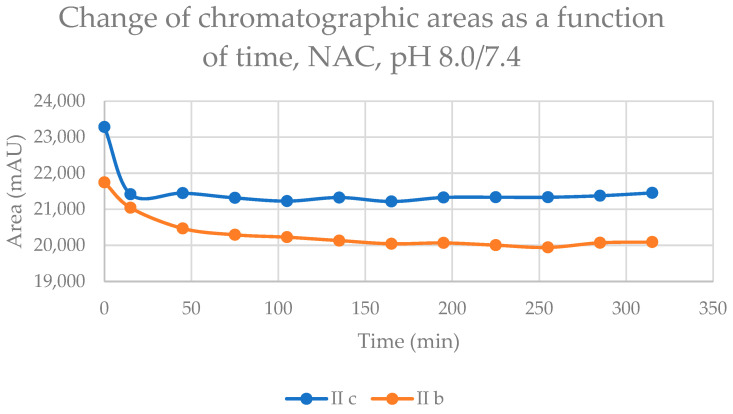
Change in the chromatographic peak area of chalcones **IIb** and **IIc** in the chalcone–NAC incubations at pH 8.0/7.4.

Initially, we investigated the reactions of **IIb** and **IIc** under basic conditions. Considering the pKa values of GSH (8.83) and NAC (9.52), about 3.6% of the GSH and 0.75% of the NAC molecules are under pH 7.4 conditions. The reaction with GSH (Figure 2) and NAC (Figure 3) of both cyclic chalcones have intrinsic reactivity with the investigated thiols. By the end of the incubation period (315 min) with GSH, the initial area of the HPLC peak corresponding to the parent compounds **IIb** and **IIc** reduced by 43.5% and 26.3 %, respectively (Table 2). While the compounds were incubated with NAC, the respective figures were 7.9% and 7.6% (Table 3). Changes in the chromatographic peak areas of the starting chalcones as a function of the incubation time indicated that the compositions reflect the equilibrium only in the case of the NAC incubation (Figure 3). The overlayed HPLC-UV chromatograms obtained by analysis of the **IIb** with GSH and NAC (Figures S1 and S2), and **IIc** with GSH and NAC (Figures S3 and S4) at the different timepoints are shown on Figures S1–S4.

As a result of the addition reactions, two new chiral centers are formed. Considering the inherent chirality of the two thiols, the formation of four diastereomeric adducts was expected. However, only two separate peaks could be detected under the present chromatographic conditions. The analysis showed a slight excess of the less polar diastereomers in both cases. The structure of the parent chalcones and their GSH and NAC conjugates were verified by HPLC-MS (Figures S5–S10). The exact mass of **IIb**, **IIc**, and the **IIb-GSH**, **IIb-NAC**, **IIc-GSH** and **IIc-NAC** adducts are summarized in Table S1.

**Figure 4:**
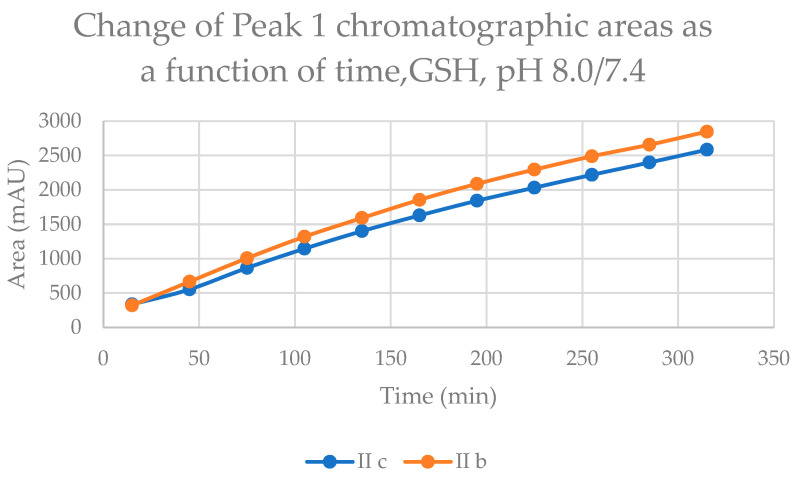
Change in the chromatographic peak area of adduct 1 of **IIb** and **IIc** in the chalcone–GSH incubations at pH 8.0/7.4.

**Figure 5:**
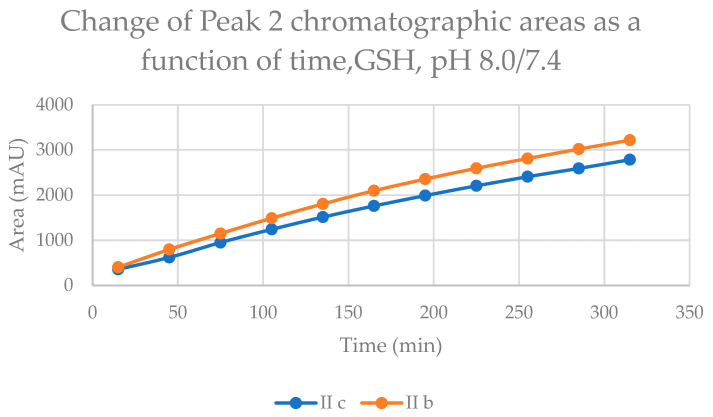
Change in the chromatographic peak area of adduct 2 of **IIb** and **IIc** in the chalcone–GSH incubations at pH 8.0/7.4.

**Figure 6:**
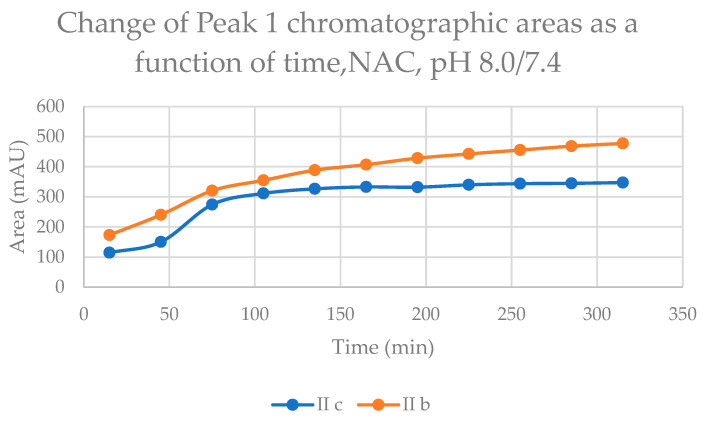
Change in the chromatographic peak area of adduct 1 of **IIb** and **IIc** in the chalcone–NAC incubations at pH 8.0/7.4.

**Figure 7:**
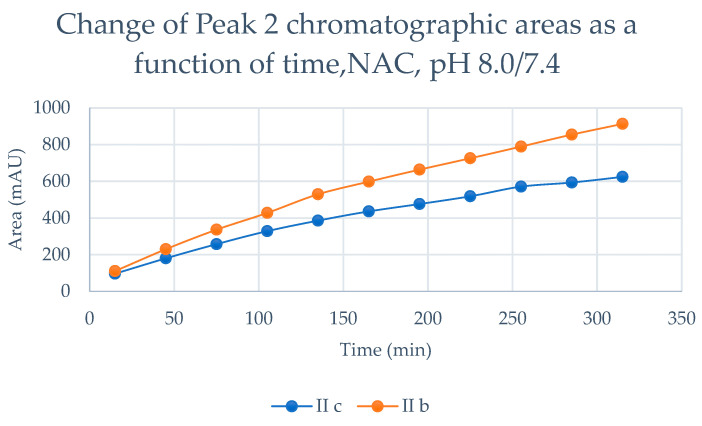
Change in the chromatographic peak area of adduct 2 of **IIb** and **IIc** in the chalcone–NAC incubations at pH 8.0/7.4.

The time course of increase in the two separated peaks, GSH-1 (Figure 4) and GSH-2 (Figure 5), and NAC-1 (Figure 6) and NAC-2 (Figure 7) showed some characteristic differences. In the case of the GSH adducts of **IIb** and **IIc**, the peak areas almost linearly increased over time. The progression curves of formation of the adducts, however, showed somewhat different slopes, especially from the 105 min timepoint (Figure 4 and Figure 5). In the case of the NAC-adducts, the progression curves deviate from linearity. The curvatures of the concave curves are different from the 75 min timepoint (Figure 6 and Figure 7) By the end of the incubation period (315 min), the ratio of the two peaks of the GSH incubations remained close to unity (1.13 and 1.08 for **IIb** and **IIc**, respectively). In the case of the NAC incubations, the respective ratios were 1.91 and 1.80 (Table 2 and Table 3). The formation of (*Z*)-chalcones could be detected in all four incubations. In the case of the NAC incubations, the area of the (*Z*)-peaks is comparable with those of the chalcone–NAC adducts (Table 2 and Table 3).

### 2.2. Reaction under Slightly Acidic (pH 6.3/6.8) Conditions

**Figure 8:**
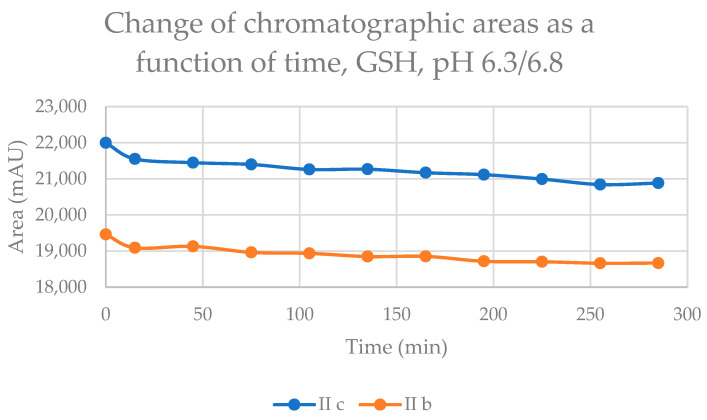
Change in the chromatographic peak area of chalcones **IIb** and **IIc** in the chalcone–GSH incubations at pH 6.3/6.8.

**Figure 9:**
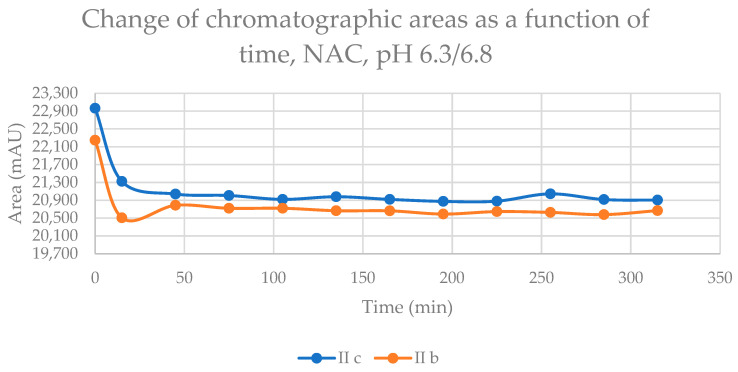
Change in the chromatographic peak area of chalcones **IIb** and **IIc** in the chalcone–NAC incubations at pH 6.3/6.8.

The reaction of the cyclic chalcones with the two thiols under slightly acidic conditions (virtual pH 6.8) mimics the cellular milieu of the cancer cells [27]. Under such conditions, about 0.9% of the GSH molecules and 0.2% of the NAC molecules exist in the more reactive thiolate form. The change in the concentration (chromatographic peak areas) of the starting chalcones **IIb** and **IIc** shows parallelism in both reactions (Figure 8 and Figure 9). By the end of the incubation period (315 min) with GSH, the initial area of the HPLC peak of **IIa** and **IIb** was reduced by 16.1% and 9.1%, respectively. While the compounds were incubated with NAC, the respective figures were 8.9% and 7.1%. These latter figures are very close to those obtained under slightly basic conditions (Table 2 and Table 3).

In the GSH incubations, the separated HPLC peak areas of the **IIb-GSH** and **IIc-GSH** diastereomers increased as closely parallel over time (Figures S11 and S12). At the end of the incubation period, the ratio of the area of two separated peaks of the chalcone–GSH adducts is close to unity (1.12 and 1.10 for **IIb** and **IIc**, respectively) (Table 4). A similar tendency could be observed for the NAC-2 peak (peak with the higher retention time) area of **IIb** and **IIc** (Figure S14). At the same time, the chromatographic peak area of the NAC-1 peak of **IIc** remained practically unchanged, but that of **IIb** slightly increased (Figure S13). As a result, the ratio of the NAC-2/NAC-1 areas at the 315 timepoint was 8.57 and 6.34 for **IIb** and **IIc**, respectively (Table 4). In all four incubations, the peak areas of the (*Z*)-chalcones are comparable to those of the formed adducts (Table 4). Since the only source of the (Z) isomers under the experimental conditions is the retro-Michael reactions, it is reasonable to presume that the observed diastereomeric distributions do not reflect the results of the kinetics-controlled reactions.

### 2.3. Reaction under Acid (pH 3.2/3.7) Conditions

Under stronger acidic conditions, the thiol function of both GSH and NAC exists exclusively in protonated (neutral) form. Although the protonated thiols can act as nucleophilic reagents, their reactivity is much lower than that of their deprotonated (negatively charged) counterparts [29]. In the chalcone–GSH incubations, progression curves of the reactions (reduction in the initial area of the chalcones) showed a very slight downhill linear shape (Figure S15). At the end of the incubations, the initial values of the peak areas of **IIb** and **IIc** were reduced by 10.6% and 5.3%, respectively. At the same time, a linear increase in the peak area of the **IIb-GSH** adducts (peak 1 and peak 2) could be observed (Figures S16 and S17). The peaks corresponding to the respective **IIc-GSH** adducts could not be detected. The ratio of the **IIb-GSH** isomeric peaks (315 min timepoint) was 1.48. The areas of the respective (*Z*) isomers were much lower than in the pH 8.0 and pH 6.3 incubations (Table 2).

In the chalcone–NAC incubations, the reduction in the initial area of the chalcones showed a very slight downhill linear shape with somewhat different slopes (Figure S18). The initial peak area of **IIb** and **IIc** was reduced by 23.7% and 12.1% by the 315 min timepoint (Table 3). However, no **II-NAC** peaks could be identified. HPLV-UV analysis of the incubates showed the formation of several small peaks that were more polar than the parent **IIb** and **IIc**. HPLC-MS investigations indicated the expected adduct formation, but it was impossible to identify them in the HPLC-UV chromatograms (Figures S19 and S20). In both incubations, the formation of the (*Z*) isomers could be seen (Table 3).

### 2.4. Molecular Modeling Analysis

**Figure 10:**
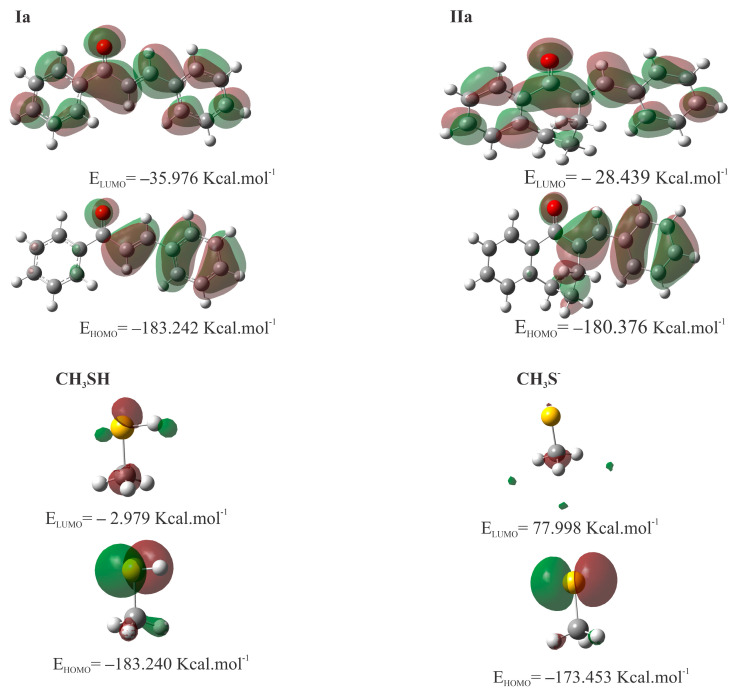
HOMO and LUMO plots for **Ia**, **IIa CH_3_SH**, and **CH_3_S^−^** calculated at the M06-2X/6-311++G (d,p) level of theory.

Table 5 shows the calculated values for molecular properties of **Ia**, **IIa**, methanethiol (**CH_3_SH**), and deprotonated methanethiol (**CH_3_S^−^**). The highest occupied molecular orbital energy (*E*_HOMO_) reflects the ability of a molecule to donate electrons, the lowest unoccupied molecular orbital energy (E_LUMO_) demonstrates the ability to accept electrons (see Figure 10), and the gap energies (ΔE_LUMO-HOMO_) are related to the chemical stability of molecules.

The chemical potential, chemical hardness, and electrophilicity are defined as ($\mu ={\left(\frac{\partial E}{\partial N}\right)}_{\upsilon }$), ($\eta =\frac{1}{2}{\left(\frac{\partial^{2}E}{\partial N^{2}}\right)}_{\upsilon }$), and ($\omega =\frac{\mu^{2}}{2\eta }$), respectively. η indicates the resistance of the molecule to alter its electronic density distribution and is higher for **IIa**. On the other hand, μ indicates the change in free energy when electrons are added or removed from the molecule. At the same time, ω is a measure of a molecule’s tendency to act as an electrophile. The value of ω increased for **Ia**, and μ decreased when compared to **IIa**. 

**Figure 11:**
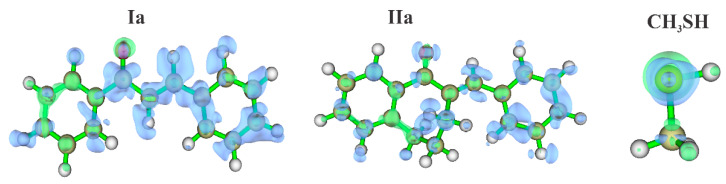
Isosurfaces of the Fukui Function, calculated at a proper value of 0.08 for the molecules **Ia**, **IIa**, **CH_3_SH**, and **CH_3_S^−^**.

Figure 11 shows the isosurfaces of the Fukui function (obtained from electron density) for the molecules **Ia**, **IIa**, and **CH_3_SH**, with a focus on atoms that can undergo nucleophilic attack (). The positive and negative regions of the Fukui function are represented by the green and blue isosurfaces, respectively.

**Figure 12:**
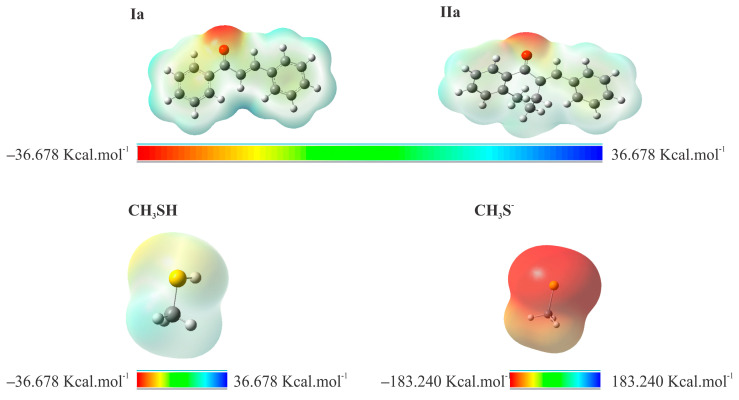
MEP surface contour of the total SCF electronic density for molecules **Ia**, **IIa**, **CH_3_SH**, and **CH_3_S^−^** at the M06-2X/6-311++G (d,p) level of theory.

In order to depict the distribution of electric charge on the molecular surface, a molecular electrostatic potential (MEP) map was generated (Figure 12). The red spots on the MEP surface represent the electron-rich sites and are susceptible to electrophilic attack. In contrast, the blue spots represent the electron-depleted regions and are sites susceptible to nucleophilic attack. For **CH_3_S^−^**, the MEP is reddish due to the −1 negative charge resulting from deprotonation.

The *k_OH_* rate constants were calculated for chalcone compounds, and the results indicate that the compounds with the highest *k_OH_* values point to greater reactivity. The order of reactivity potential was observed as **CH_3_SH** (9.15 × 10^9^ M^−1^ s^−1^) **Ia** (9.01 × 10^9^ M^−1^ s^−1^) > **IIa** (7.85 × 10^9^ M^−1^ s^−1^) > **CH_3_S^−^** (5.48 × 10^9^ M^−1^ s^−1^).

## 3. Discussion

Our experiment showed that both cyclic chalcone analogs (**IIb** and **IIc**) have intrinsic reactivity with GSH and NAC under all three experimental conditions. The results strengthen the results of our previous studies obtained by TLC analysis of similar incubations with GSH of the two compounds [13]. Considering the pKa values of GSH (8.83) and NAC (9.52) thiols, it can be seen that the fraction of the stronger nucleophile thiolate form of GSH is higher than that of NAC under each experimental condition. Under the slightly basic conditions (pH 8.0/7.4), the rate of reduction in the HPLC peak area of the starting chalcones showed a linear decrease. Since the area is based on the absorbance (logarithmic function of the concentration) of the compounds, the reactions follow pseudo-first-order kinetics. In the case of both thiols, relatively high amounts of (*Z*)-chalcone isomers could be detected in the incubations (Table 2 and Table 3). Since the reaction mixtures were incubated in the dark, the corresponding retro-Michael reactions are the only source of the (Z)-isomer formations. Accordingly, the progression curves of the incubations (Figure 2 and Figure 3) reflect the disappearance of the starting compounds due to the net change in the reversible reactions. Similar levels of the respective (*Z*)-isomers could be detected in the incubations performed under slightly acidic (pH 6.3/6.7) conditions (Table 2 and Table 3). On the contrary, the reactions of the respective open-chain chalcones (**Ib** and **Ic**) performed under identical conditions did not result in a detectable level of (*Z*)-isomers in the GSH or the NAC incubations [24].

The results obtained in the pH 6.3/6.7 incubations are similar to those of the pH 8.0/7.4 ones (Table 2 and Table 3). Under such conditions, however, the composition of both incubations represents the equilibrium mixtures. Similar to the pH 8.0/7.4 incubations, the conversion of **IIb** is somewhat higher in the case of both thiols. The observation further strengthens the previously suggested view that the different reactivities can be (at least partly) the result of the different stability of the thiol adducts [24,30]. Similar to the results obtained under identical conditions with the respective open-chain chalcones (**I**), the 4-methyl-substituted derivative (**IIb**) forms the more stable adducts.

Comparing the compositions of the 315 min incubation mixtures of the two series (**I** and **II**), it can be seen that the conversions of the 4-CH_3_- and 4-OCH_3_-substituted chalcones (**Ib** and **Ic**) are much higher than those of the **IIb** and **IIc** (Table 6). ^13^C NMR shifts, indicating the electron density around the particular nucleus, of the β-carbon atom of **IIb** (138.0 ppm) and **IIc** (137.7 ppm), were reported to be similar. A similar slight (0.3 ppm) difference was observed in the case of the respective open-chain chalcones **Ib** and **Ic** [31]. Since the nature of thiols and the aromatic substituents are the same, the ring structure can explain the observed differences in reactivities of the two series.

Amslinger et al. investigated the thiol reactivity of chalcones with various substituents in their α-position. The kinetics of thiol reactivities of the derivatives were correlated with some of their biological effects directly connected to their Michael acceptor ability [32,33]. For example, α-methyl substitution of 2’,3,4,4′-tetramethoxychalcone (TMC) decreased, α-cyano substitution substantially increased the thiol reactivity of the nonsubstituted TMC [34]. Based on these earlier observations, it is reasonable to suppose that the reduced reactivity of the benzosuberone derivatives **IIb** and **IIc** is the consequence of added effects of the α-alkyl substitution and the conformational strain caused by the cyclic structure of the starting enone and the reaction intermediate. Further research is needed to characterize the electronic and stereochemical effects of the ring numerically.

As a result of the addition reactions, the formation of four diastereomeric adducts is possible. Because of the inherent chirality of GSH and NAC, two *cis* adducts and two *trans* adducts are expected to be formed. Earlier, Armstrong et al. reported on the stereochemistry of the GSTM 4-4-catalyzed reaction of GSH and the open-chain chalcone analog (*E*)-(4′-X-phenyl)-3-butene-2-ones (PBO). In the reactions, a higher amount of the more polar adducts were formed [35]. Based on the results of HPLC separation of the diastereomeric pairs of the PBO-GSH [35] and the **I**-GSH [24] adducts, we can presume that the two separated peaks formed in the present reactions correspond to the diastereomeric *cis* and *trans* adducts.

The ratio of the area of the two separated peaks in the GSH incubates (315 min timepoint) was close to the unity for **IIb** and **IIc** under both pH 8.0/7.4 and 6.3/6.8 conditions (Table 2). Similar to our previous results, higher peak areas of the least polar adducts were observed in each case. On the contrary, HPLC analysis of the reactions of **IIb** and **IIc** with NAC showed different (1.8–8.57 times) excess of the least polar diastereomer (Table 3). Similar to the previous results obtained with the open-chain chalcones (**Ib** and **Ic**) [24], the observed diastereoselectivity was affected by the nature of the 4-substituent and the pH. Thus, the methyl–substituted **IIb** showed higher diastereoselectivity at both pH values. Diastereoselectivity was increased as the pH was reduced (Table 3). It is worth mentioning, however, that the observed diastereoselectivities do not reflect the diastereoselectivity of the addition reactions. Under both conditions (pH 8.0/7.4 and 6.3/6.8), the peak areas of the (*Z*)-isomers and the adducts are comparable (Table 3). Since the retro-Michael reaction is the only source of formation of the (*Z*)-isomers, the observed ratios reflect the actual balance of the kinetic and thermodynamic controls.

Under the acid conditions (pH 3.2/3.7), the formation of the respective conjugates is exclusively due to the nucleophilic addition of the protonated thiol forms onto the polarized carbon-carbon double bounds. In comparison of the respective compositions of the GSH incubates with those of the previously reported (open chain) chalcones (**Ib** and **Ic**) [24], the derivatives with the same substituent showed similar GSH reactivities (Table 2). However, different results were obtained in the case of the reactions with NAC. The 315min percent conversion (reduction in the initial peak area) was found to be higher for **IIb** (23.7%) and **IIc** (12.1%) than those of the corresponding open-chain chalcones **Ib** and **Ic** (10.9% and 1.5%, respectively (Table 6). However, no **II-NAC** adducts could be identified in the HPLC-UV chromatograms. Instead, several small, unidentified peaks appeared (Figures S19 and S20). HPLC-MS analysis could identify the expected conjugates. The structural characterization of the other products is out of the scope of the present work.

To obtain physicochemical properties insights into different reactivities of chalcones (**I**) [24] and their seven-membered cyclic analogs (**II**), HOMO and LUMO molecular orbital energy and some electrophilic reactivity parameters of **Ia**, **IIa**, and as model thiols **CH_3_SH** and **CH_3_S^−^** were calculated (Table 5). According to the Hard and Soft, Acids and Bases (HSAB) theory [36], nucleophilic-electrophilic reactions occur preferably between electrophiles and nucleophiles of similar hardness or softness. In the case of the α,ß-unsaturated ketone, the carbonyl oxygen atom withdraws electrons from the C_2_=C_10_ bond—generating an electron deficiency at C_10_—the most likely site to receive nucleophilic attacks. In methanethiol, the electrophilic attacks can occur at the sulfur atom. In compounds **Ia** and **IIa**, the carbonyl O has a high negative charge density, indicating its Lewis base behavior. On the other hand, regions of lower charge density, which appear in blue, indicate the Lewis acid behavior of the molecules.

The LUMO energy showed that **Ia** (−35.98 kcal/mol) is more acidic than **IIa** (−28.44 kcal/mol. The HOMO energy of **CH_3_SH** is (−183.240 kcal/mol). It increases to (−173.453 kcal/mol) in the deprotonated form (**CH_3_S^−^**) indicating its higher nucleophilic reactivity. These characters are also reflected by all of the other determined parameters (Table 5). Therefore, molecular orbital calculations provided data to support the experimental findings. The equilibrium (close-to-equilibrium) compositions of **Ib** and **Ic** show a higher product ratio than the cyclic chalcone analog **IIb** and **IIc**.

## 4. Materials and Methods

### 4.1. Chemicals and Reagents

Chalcones **IIb** and **IIc** were synthesized as previously published [10]. Their structures were characterized by IR and NMR spectroscopy [37]. The purity and structures of the investigated samples were verified by TLC, melting point, and HPLC-MS (Figures S5 and S6). Reduced l-glutathione, N-acetyl l-cysteine, HPLC, and MS-grade methanol solvent were obtained from Sigma-Aldrich (Budapest, Hungary). Trifluoroacetic acid HiperSolve CHROMANORM and formic acid were obtained from VWR (Budapest, Hungary) and Fischer Scientific (Budapest, Hungary), respectively. Deionized water for use in HPLC and HPLC-MS measurements was purified by Millipore Direct-Q^TM^ (Merck Life Science, Budapest, Hungary), at the Institute of Pharmaceutical Chemistry (University of Pécs, Pécs, Hungary). Mobile phases used for HPLC measurements were degassed by an ultrasonic water bath before use.

### 4.2. Preparation of Solutions

The thiol solutions (reduced glutathione (GSH) and *N*-acetylcysteine (NAC)) preparation were as follows: 2.0 × 10^−1^ mol·L^−1^ (0.3 mmol) of the respective thiol was dissolved in water, and the pH was set to either 3.2, 6.3, or 8.0 using 1M NaOH solution to a final volume of 1.5 mL (solution-1). The chalcone solution consisted of 6.5 × 10^−3^ mol·L^−1^ (0.03 mmol) chalcone analog dissolved in 4.6 mL HPLC-grade methanol (solution-2). Solution-1 and solution-2 were mixed to give a final volume of 6.1 mL. The molar ratio of thiol to chalcone in the mixture was 10:1. The mixture was kept in the dark, 37 °C water bath for 315 min. The first sample was taken at 15 min, and onward samples were taken at every 30 min time points (11 samples in total).

To evaluate the initial (0 min) peak area of chalcones **IIa** and **IIb**, solution-2 was prepared without any change, while solution-1 was prepared without the thiol component. Before mixing, the solutions were pre-incubated at 37 °C for 30 min to mimic the incubation conditions. To compare the products of the previously proven light-initiated *E/Z* isomerization of the parent compounds [27] with those of the non-light (retro-Michael addition)-initiated isomerization, solution-2 of the respective chalcones were prepared and exposed to the unscattered laboratory light for 1 week. The solutions were analyzed by HPLC-UV-VIS and HPLC-MS. (Figures S21 and S22).

### 4.3. RP-HPLC-UV-VIS Measurements

UV–VIS detector coupled Agilent 1100 HPLC system analyzed the samples at 260 nm wavelength. The separation system was a reversed-phase chromatographic system, and the column Zorbax Eclipse XBD-C8 (150 mm × 4.6 mm, particle size 5 µm; Agilent Technologies, Waldbronn, Germany) was used. The oven temperature was set to 25 °C to avoid room temperature fluctuations. The injection volume was 10 µL. At a 1.2 mL/min flow rate, gradient elution was performed by (A) water and 0.1% trifluoroacetic acid and (B) methanol and 0.1% trifluoroacetic acid. The elution profile consisted of 8 min of 40% isocratically, an increase to 60% B in 4 min, and a further linear increase of eluent B to 90% in 3 min. The elution gradient remained constant for a 5 min period. Then it was linearly decreased to the initial 40% in 2 min, followed by a 3 min constant of 40% of eluent B for equilibration of the column. 

### 4.4. HPLC-MS Measurements

HPLC ESI-MS analyses were performed on an Ultimate 3000 liquid chromatograph (Dionex, Sunnyvale, CA, USA) coupled with a Thermo Q Exactive Focus quadrupole-Orbitrap hybrid mass spectrometer (Thermo Fisher Scientific, Waltham, MA, USA). The scan monitored *m/z* values ranging from 100 to 1000 Da. Data acquisition was carried out using Q Exactive Focus 2.1 and Xcalibur 4.2 software (Thermo Fisher Scientific). Analysis of compounds and adducts was performed in HESI positive and negative ionization modes with the following parameters: spray voltage, 3500 V; vaporizer temperature, 300 °C; capillary temperature, 350 °C; spray and auxiliary gas flows, 30 and 10 arbitrary units, respectively; resolution, 35,000 at 200 *m/z*; and fragmentation, 20 eV.

HPLC separation was performed on an Accucore C18 column (150 mm × 2.1 mm, particle size 2.6 µm), and an Accucore C18 guard column (5 mm × 2.1 mm, particle size 2.6 µm) was also used. The injection volume was 5 µL; the flow rate was 0.4 mL/min. Data analysis and evaluations were performed using Xcalibur 4.2 and FreeStyle 1.7 software. A binary gradient of eluents was used, consisting of mobile phases A and B.

The gradient parameters in chalcones were (A) water and 0.1% formic acid and (B) methanol and 0.1% formic acid. The gradient elution was as follows: isocratic elution for 1 min to 20% eluent B, continued by a linear gradient to 100% in 9 min, followed by an isocratic plateau for 2 min. Then, the column was equilibrated back to 20% in 0.5 min and continued isocratically for 2.5 min. The sampler was at room temperature and the column oven was at 40 °C.

The parameters of the gradient in the case of adducts were (A) water and 0.1% formic acid and (B) methanol and 0.1% formic acid. The gradient elution was as follows: isocratic elution for 1 min to 10% eluent B, continued by a linear gradient to 95% in 13 min, followed by an isocratic plateau for 3 min. Finally, the column was equilibrated to 10% in 0.1 min and continued isocratically for 2.9 min. The sampler was at room temperature, and the column oven was at 40 °C. The diode array detector was also set at 260 nm wavelength alongside MS analysis.

### 4.5. Molecular Modeling Analysis

The structures **Ia**, **IIa**, **CH_3_SH**, and **CH_3_S^−^** were constructed using the Gaussview 6.0 software. Theoretical calculations were performed by DFT [38,39], implemented in the G16 [40] software package. The molecules were optimized using the hybrid exchange and correlation functional with long-range correction, M06-2X [41], combined with the basis set 6-311++G(d,p) in the gas phase. Frontier molecular orbitals (FMO) [42] were obtained. Molecular electrostatic potential maps contributed to the global electrophilicity analysis through their electronic isodensity surfaces. MEP [43] maps provide a visual representation of the electrostatic potential on the surface of a molecule, which can reveal regions of high and low electron density. The electrostatic potential V(**r**) [44] at point **r** is defined as.

(1) $$V\left(r\right)=\underset{\alpha }{\sum }\frac{Z_{A}}{\left|r_{\alpha }-r_{A}\right|}-\int \frac{\rho \left(r\right)}{r_{\alpha }-r}\mathrm{dr}$$

where Z*_A_* is the charge of nuclei *a* at point **r***a* and $\rho \left(r\right)$ is the charge density at point **r**. The local electrophilicity of the molecules was determined by the Fukui function [45,46], and then it was possible to predict the molecular site selectivity.

(2) $$f\left(r\right)={\left[\frac{\partial \rho \left(r\right)}{\partial N}\right]}_{v}$$

where N is the number of electrons in the system, and the constant term v in the partial derivative is external potential. Multiwfn 3.6 program [47] was used to calculate the Fukui. In addition, the *py*SiRC [48], a machine-learning computational platform, was used to simulate oxidation reactions facilitated by free-radical compounds. To imitate the oxidation impact induced by a radical attack, the hydroxyl radical (OH) was chosen as the archetype system of degradation reactions. The reaction rate constant of the oxidative attack caused by the hydroxyl radical on chalcones compounds was predicted using the XGBoost ML algorithm and the MACCS fingerprint was employed as a structural descriptor.

## 5. Conclusions

The present work investigated the instantaneous GSH- and NAC-reactivity of two cyclic chalcones analogs (**IIb** and **IIc**) with different cancer cell cytotoxicities. The reactivity of the two compounds was investigated under three different acid-base conditions. The progress of the reactions, disappearance of the parent compounds, and appearance of the thiol-conjugates were monitored by the HPLC-UV method. Comparison of the reactivities of **IIb** and **IIc** with those of the respective open-chain chalcones showed the cyclic analogs to be less reactive under each investigated conditions. In both series, the 4-CH_3_ substituted derivatives displayed higher reactivities. The substituent effect could be rationalized by the higher electron-donating power of the 4-OCH_3_ substituent. The alkyl substitution of the α-carbon atom and the cyclic structure can explain the lower reactivities of **IIb** and **IIc** compared to the open-chain counterparts (**Ib** and **Ic**). The theoretical calculations of orbital energies and electrophilic reactivity parameters provided evidence that supports the experimental findings—the open chalcone exhibits higher electronegativity and reactivity than its cyclic counterpart.

Cytotoxicity and cell cycle modulating effects of **IIb** and **IIc** showed characteristic differences. The present results do not indicate a direct correlation with the cancer cell cytotoxicity of the two derivatives, which are of two orders of magnitude different against most of the investigated cell lines. The anticancer potential of chalcones is correlated with their ability to act on various molecular targets such as ABCG2, tubulin, activated nuclear B cell growth (NF-κB), vascular endothelial growth factor (VEGF), tyrosine kinase receptor (EGFR), mesenchymal-epithelial transition factor (MET), 5-α reductase, ACP-reductase, histone deacetylase, p53, CDC25B (protein tyrosine phosphatase), retinoic acid receptors, estrogenic topoisomerase receptors and MDM2 [9]. Considering the present and our previous results [10,11,12,13,14,15,16], it is reasonable to suppose that the molecular basis of the different biological effects of **IIb** and **IIc** is related to the non-covalent interactions of the compounds [49].

## Figures and Tables

**Table 1:** IC_50_ (μM) data of selected *E*-2-(4′-X-benzylidene)-1-benzosuberones (**II**) [10].

Compound	P388	L1210	Molt 4/C8	CEM	Human Tumor Cells
**IIa**	12.7	106.0	42.7	28.9	18.6
**IIb**	11.8	25.0	21.3	11.4	11.2
**IIc**	1.6	0.34	0.47	0.35	0.27

**Table 2:** Retention times (t_R_) ^1^ and integrated peak areas (A) of the investigated cyclic chalcone analogs (**IIb** and **IIc**) and their GSH adducts ^2^.

pH ^3^	Compound	t_R_ (*E*)-Chalcone	Area Ratio ^4^ A_315_/A_0_	t_R_ (*Z*)-Chalcone	Area (*Z*)-Chalcone	t_R_ GSH–1	Area GSH–1	t_R_ GSH–2	Area GSH–2
3.2	**IIb**	17.1	0.89	16.8	55.1	14.8 ^5^	74.9	15.2 ^5^	111.5
3.2	**IIc**	16.6	0.95	16.3	136.1	ND ^5^	-	ND ^5^	-
6.3	**IIb**	17.0	0.84	16.7	446.6	14.6	297.2	15.1	331.8
6.3	**IIc**	16.9	0.91	16.7	513.4	14.2	233.6	14.8	256.4
8.0	**IIb**	17.4	0.57	17.1	302.8	15.0	2847.0	15.4	3216.3
8.0	**IIc**	16.8	0.74	16.5	412.0	13.9	2584.9	14.6	2785.0

^1^ Retention times in minutes; ^2^ data refer to the average of two independent measurements at the 315 min time point; ^3^ pH value of the aqueous thiol solution; ^4^ ratios of peak areas measured at 0 and 315 min; ^5^ not detectable.

**Table 3:** Retention times (t_r_) ^1^ and integrated peak areas (A) of the investigated cyclic chalcone analogs (**IIb** and **IIc**) and their NAC adducts ^2^.

pH ^3^	Compound	t_R_ (*E*)-Chalcone	Area Ratio ^4^ A_315_/A_0_	t_R_ (*Z*)-Chalcone	Area (*Z*)-Chalcone	t_R_ NAC–1	Area NAC–1	t_R_ NAC–2	Area NAC–2
3.2	**IIb**	17.1	0.76	16.8	124.1	N/D ^5^	-	N/D ^5^	-
3.2	**IIc**	16.6	0.88	16.3	126.9	N/D ^5^	-	N/D ^5^	-
6.3	**IIb**	17.5	0.93	17.2	118.9	16.3	60.0	16.5	513.9
6.3	**IIc**	16.7	0.91	16.4	184.5	15.3	61.8	15.6	392.1
8.0	**IIb**	17.5	0.92	17.2	467.5	16.3	477.7	16.5	913.4
8.0	**IIc**	17.0	0.92	16.8	541.9	15.7	347.5	15.9	624.2

^1^ Retention times in minutes; ^2^ data refer to the average of two independent measurements at the 315 min time point; ^3^ pH value of the aqueous thiol solution; ^4^ ratios of peak areas measured at 0 and 315 min, ^5^ not detectable.

**Table 4:** HPLC-UV areas of the (*Z*) isomers and the diastereomeric NAC-1 and NAC-2 peaks in the NAC-incubation of **IIb** and **IIc** under slightly acidic (pH 6.3/6.7) conditions.

Compound	Time (Minute)	Area Z-Chalcone	Area NAC-1	Area NAC-2	Ratio Area NAC-2/NAC-1
**IIb**	75	91.8	35.3	224.1	6.3
	165	107.2	52.7	349.7	6.6
	255	114.9	56.8	448.3	7.9
	315	118.9	60.0	513.9	8.6
**IIc**	75	136.5	54.0	148.9	2.8
	165	159.4	59.5	249.3	4.2
	255	175.3	58.4	333.2	5.7
	315	184.5	61.8	392.1	6.3

**Table 5:** Reactivity indices were obtained for **Ia**, **IIa**, **CH_3_SH**, and **CH_3_S^−^** at the M06-2X/6-311++G(d,p) level of theory.

Descriptors	Ia kcal.mol^−1^	IIa kcal.mol^−1^	CH_3_SH kcal.mol^−1^	CH_3_S^−^ kcal.mol^−1^
E_HOMO_	−183.24	−180.38	−183.240	−173.453
E_LUMO_	−35.98	−28.44	−2.979	77.998
ΔE_HOMO-LUMO_	147.27	151.94	180.261	251.451
Chemical Potential (η)	−109.608	−104.405	−93.109	−47.728
Chemical Hardness (μ)	147.264	151.930	180.261	251.451
Electrophilicity Index (ω)	40.791	35.873	24.047	4.530

**Table 6:** Percent reduction in initial chalcone HPLC-UV peaks in the 315 min GSH and NAC incubation mixtures of the series **I** and **II**.

Compound	pH	Reagent Thiol	Reduction in Initial Peak Area at the 315 min Timepoint (%)	Reagent Thiol	Reduction in Initial Peak Area at the 315 min Timepoint (%)
**Ib**	8.0/7.4	GSH	96.3 *	NAC	94.8 *
**IIb**	8.0/7.4	GSH	43.5	NAC	7.6
**Ic**	8.0/7.4	GSH	92.1 *	NAC	90.2 *
**IIc**	8.0/7.4	GSH	26.3	NAC	7.9
**Ib**	6.3/6.7	GSH	90.6 *	NAC	75.6 *
**IIb**	6.3/6.7	GSH	16.1	NAC	7.1
**Ic**	6.3/6.7	GSH	78.3 *	NAC	53.3 *
**IIc**	6.3/6.7	GSH	9.1	NAC	9.0
**Ib**	3.2/3.7	GSH	19.3 *	NAC	10.9 *
**IIb**	3.2/3.7	GSH	10.6	NAC	23.7
**Ic**	3.2/3.7	GSH	4.2 *	NAC	1.5 *
**IIc**	3.2/3.7	GSH	5.3	NAC	12.1

* Calculated on the bases of data published in [24].

## Data Availability

Not applicable.

## Supplementary Materials

The supporting information can be downloaded at: https://www.mdpi.com/article/10.3390/ijms24108557/s1.

## Sample Availability

Samples of the compounds **I** and **II** are available from the authors.
