# Supplementary material for: (E)-2-Benzylidenecyclanones: Part XVIII Study the Possible Link between Glutathione Reactivity and Cancer Cell Cytotoxic Effects of Some Cyclic Chalcone Analogs A Comparison of the Reactivity of the Open-Chain and the Seven-Membered Homologs
Source: Int J Mol Sci. 2023 May 10;24(10):8557. doi: 10.3390/ijms24108557 (PMC10218606; doi:10.3390/ijms24108557)
Supplement: Supplementary file 1 [file ijms-24-08557-s001.zip › ijms-2373830-supplementary.pdf]

SUPPLEMENTARY MATERIALS

Table S1. Formula and exact mass (with abundance) of **IIb**, **IIc**, and the **IIb-GSH**, **IIb-NAC**, **IIc-GSH** and **IIc-NAC** adducts.*

Compound	Formula	Exact mass (a.m.u.)	Abundance (%)
**IIb**	$C_{19}H_{18}O$	262.1358	80.71
**IIc**	$C_{19}H_{18}O_2$	278.1307	80.51
**IIb-GSH**	$C_{29}H_{35}N_3O_7S$	569.2196	66.81
**IIb-NAC**	$C_{29}H_{35}N_3O_8S$	585.2145	66.65
**IIc-GSH**	$C_{24}H_{27}NO_4S$	425.1661	71.71
**IIc-NAC**	$C_{24}H_{27}NO_5S$	441.1610	71.54

*Calculated by the Scientific Instrument Services (SIS) calculator (https://www.sisweb.com/referenc/tools/exactmass.htm).

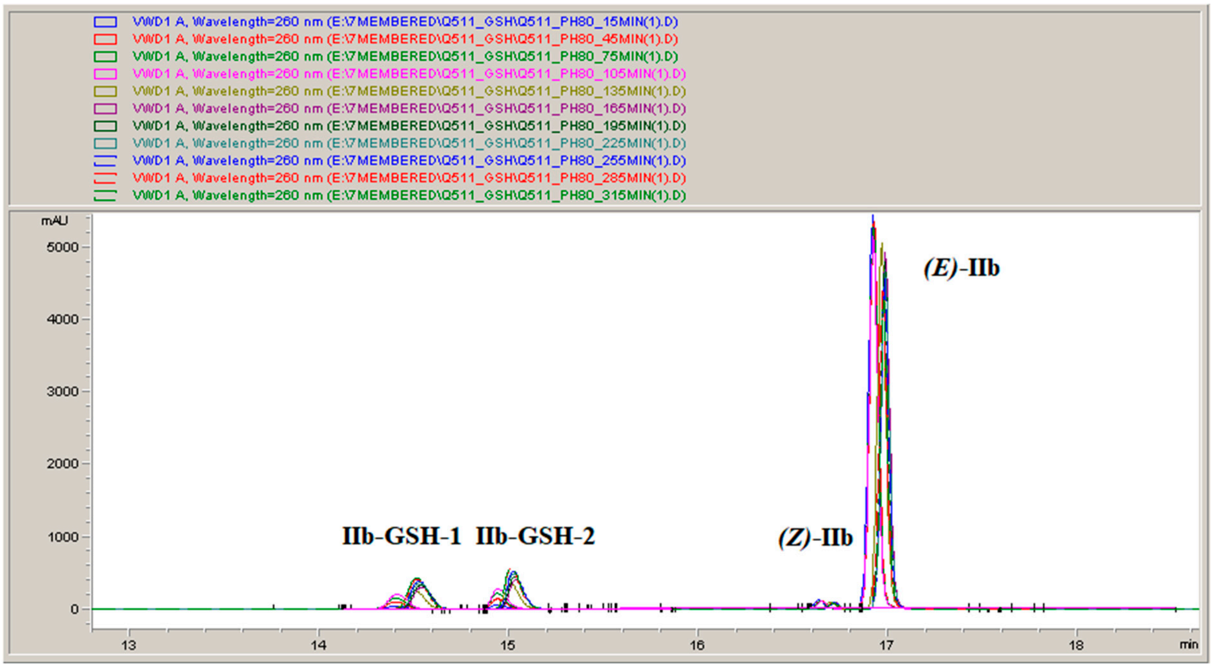

Figure S1. Overlayed HPLC-UV chromatograms of the 15, 45, 75, 105, 135, 165, 195, 225, 255, 285, and 315 min timepoint samples of the incubation mixture of **IIb** with GSH under pH 8.0/7.4 conditions.

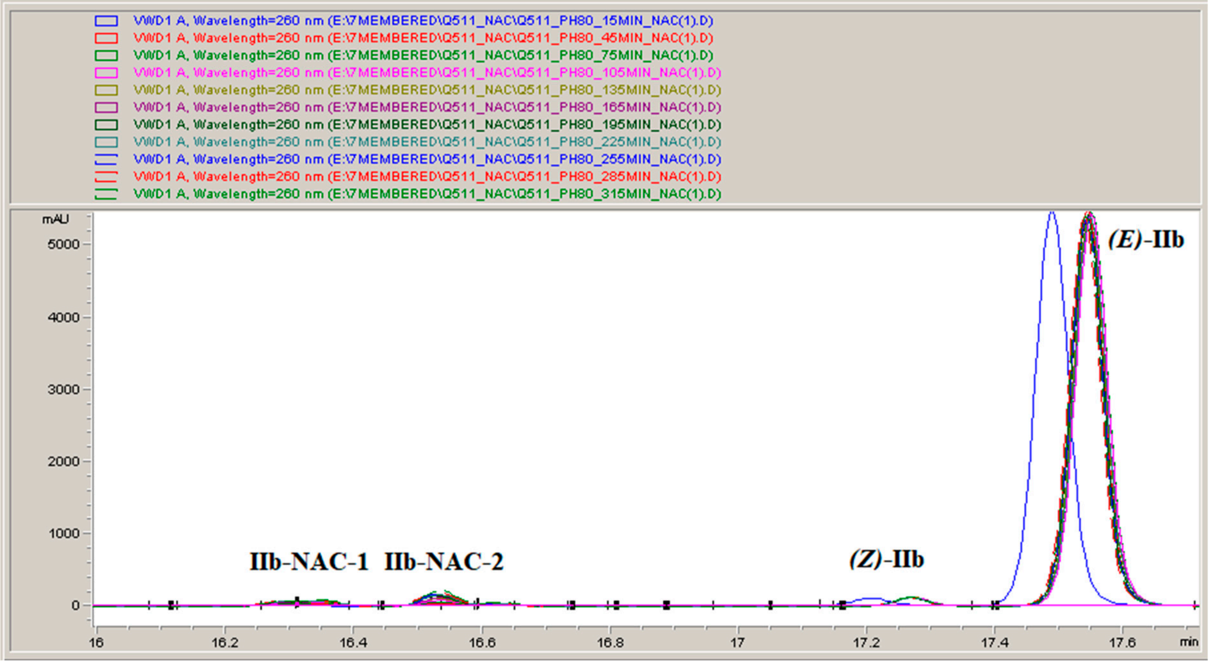

Figure S2. Overlayed HPLC-UV chromatograms of the 15, 45, 75, 105, 135, 165, 195, 225, 255, 285, and 315 min timepoint samples of the incubation mixture of **IIb** with NAC under pH 8.0/7.4 conditions. (The retention time of (*Z*)- and (*E*)-**IIb** in the 15 minute sample is shorter due to the improper conditioning of the HPLC column.)

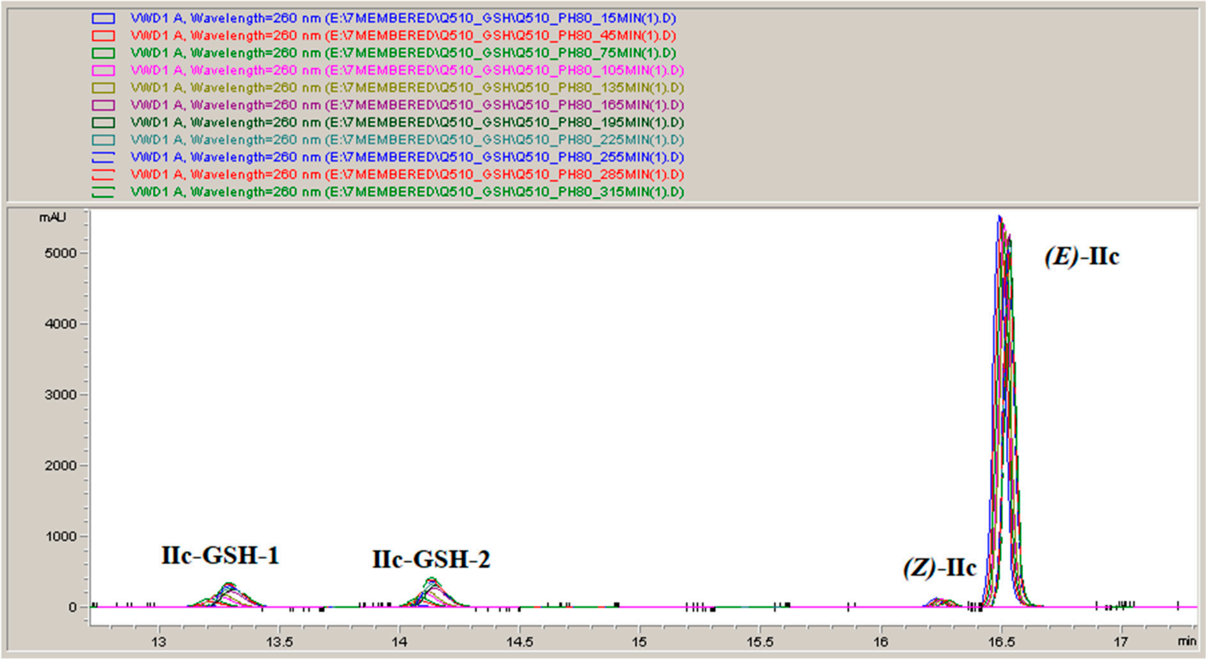

Figure S3. Overlayed HPLC-UV chromatograms of the 15, 45, 75, 105, 135, 165, 195, 225, 255, 285, and 315 min timepoint samples of the incubation mixture of **IIc** with GSH under pH 8.0/7.4 conditions.

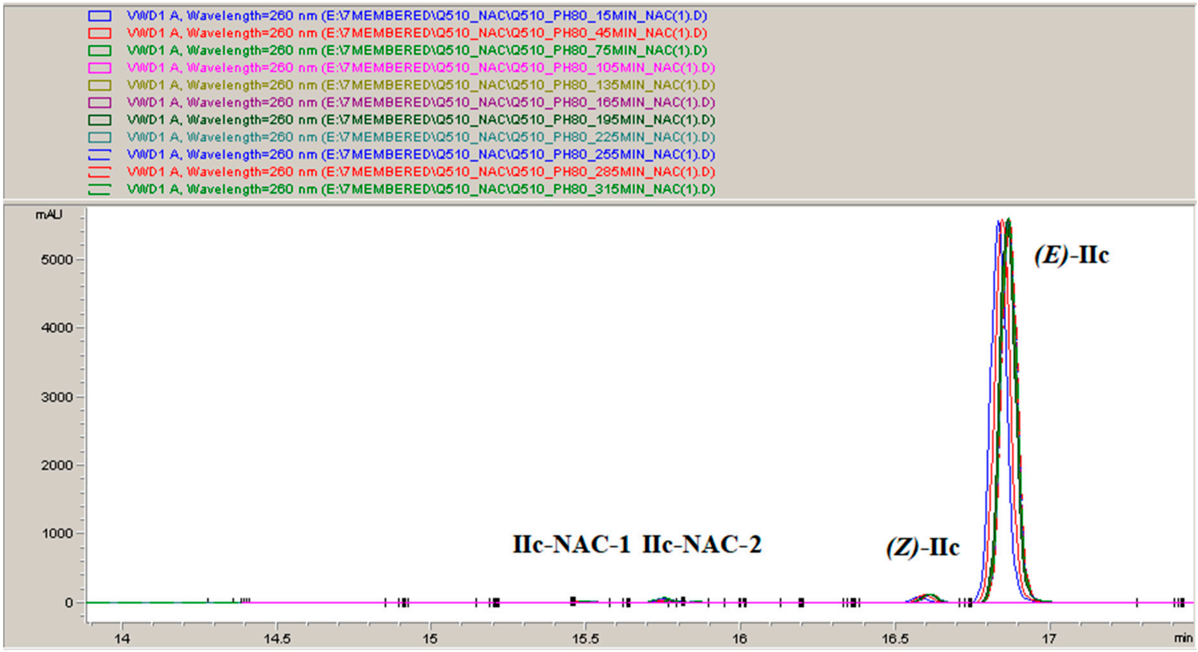

Figure S4. Overlayed HPLC-UV chromatograms of the 0, 15, 45, 75, 105, 135, 165, 195, 225, 255, 285, and 315 min timepoint samples of the incubation mixture of **IIc** with NAC under pH 8.0/7.4 conditions.

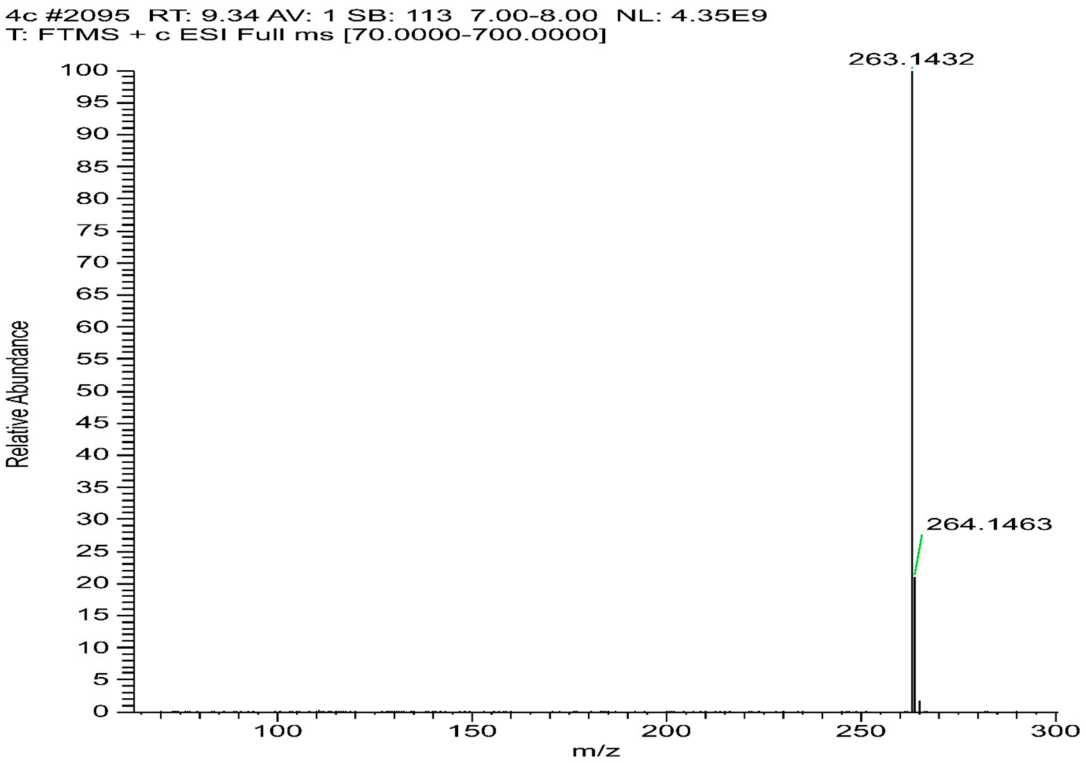

Figure S5. High-resolution, positive-mode HESI MS spectrum of **IIb**.

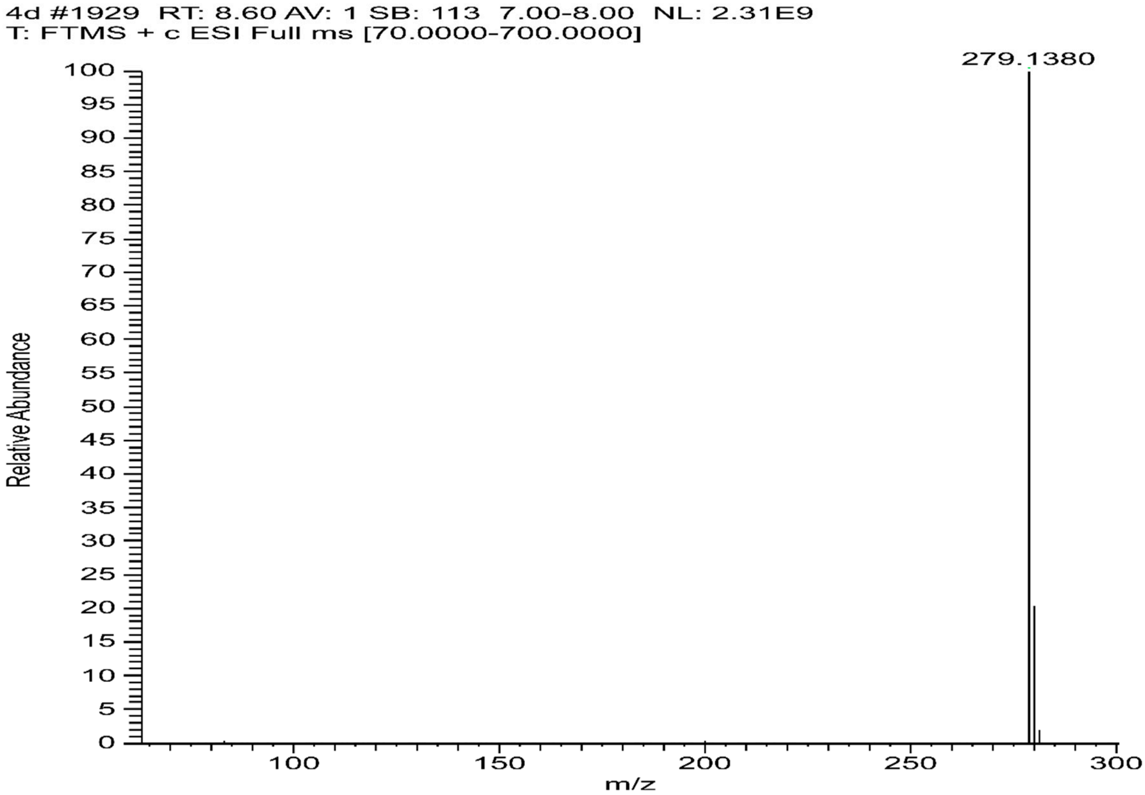

Figure S6. High-resolution, positive-mode HESI MS spectrum of **IIc**.

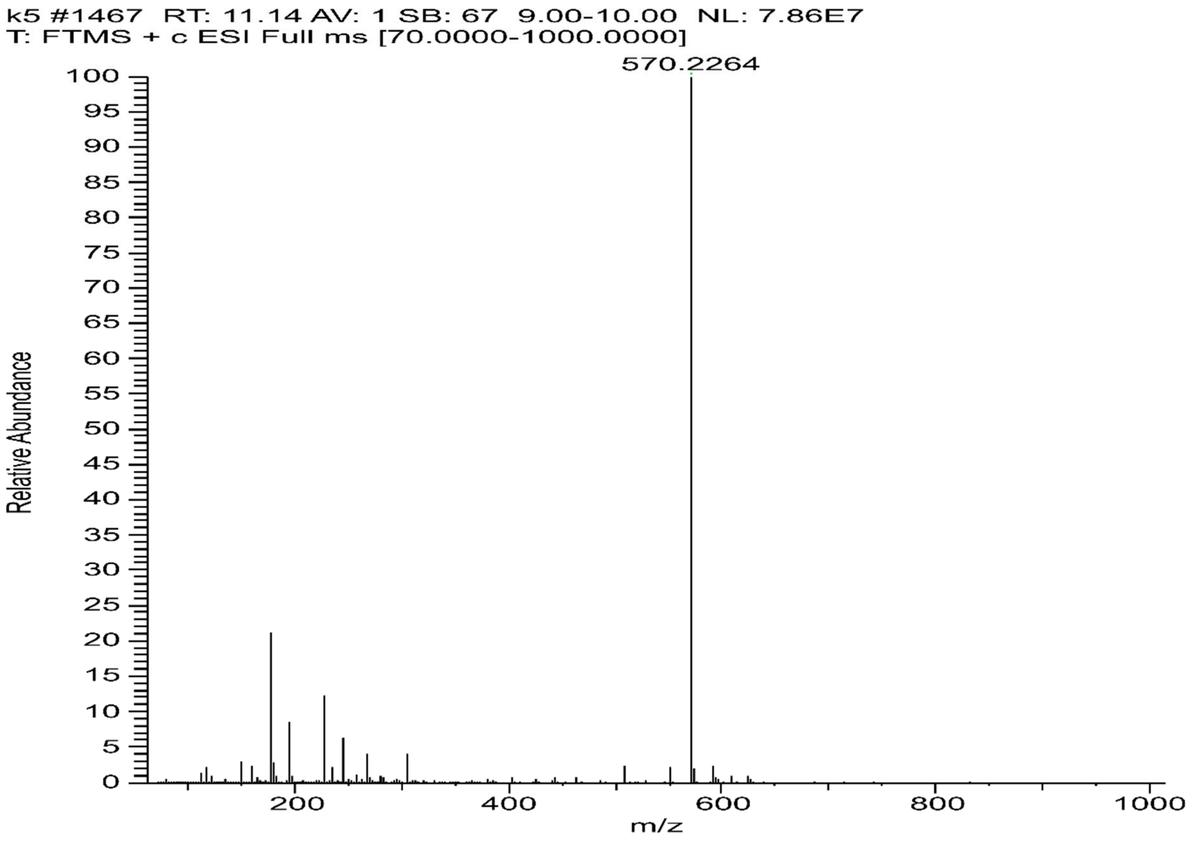

Figure S7. High-resolution, positive-mode HESI MS spectrum of **IIb–GSH** conjugate.

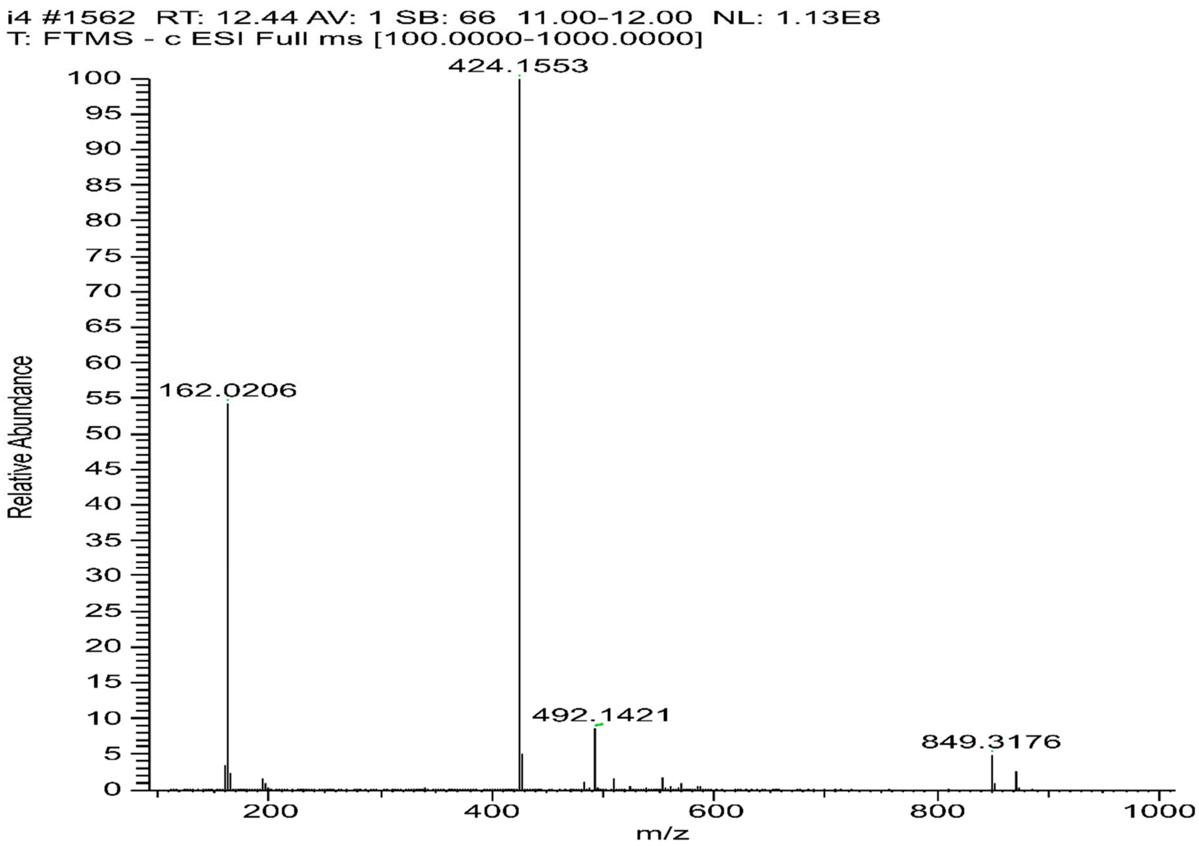

Figure S8. High-resolution, negative-mode HESI MS spectrum of **IIb–NAC** conjugate.

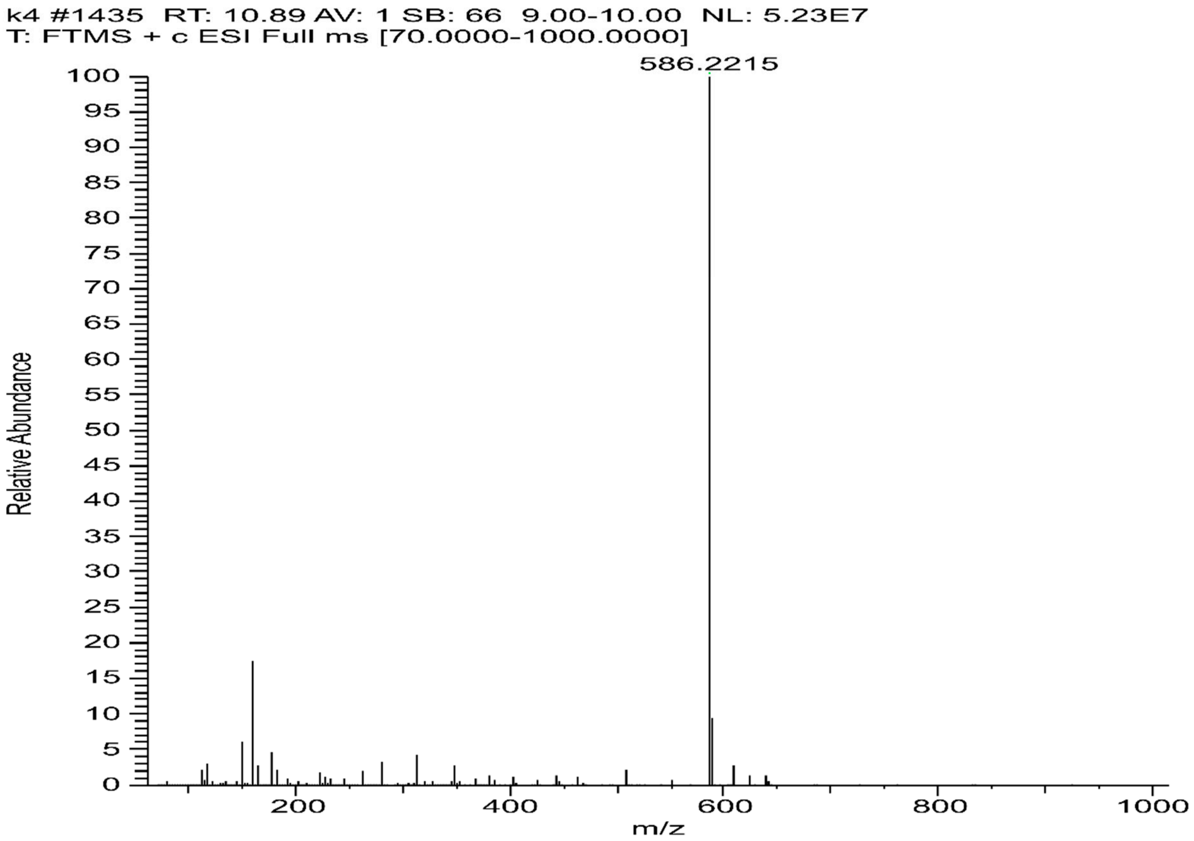

Figure S9. High-resolution, positive-mode HESI MS spectrum of **IIc–GSH** conjugate.

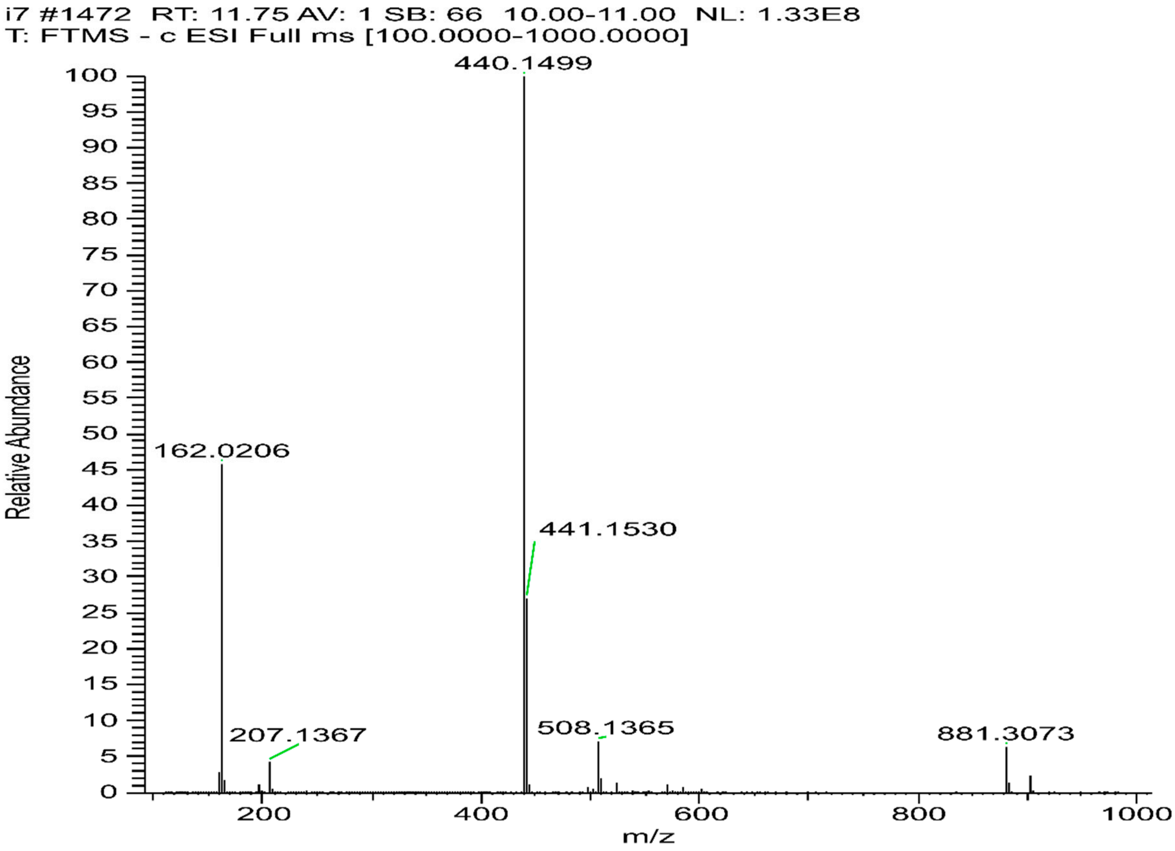

Figure S10. High-resolution, negative-mode HESI MS spectrum of **IIc–NAC** conjugate.

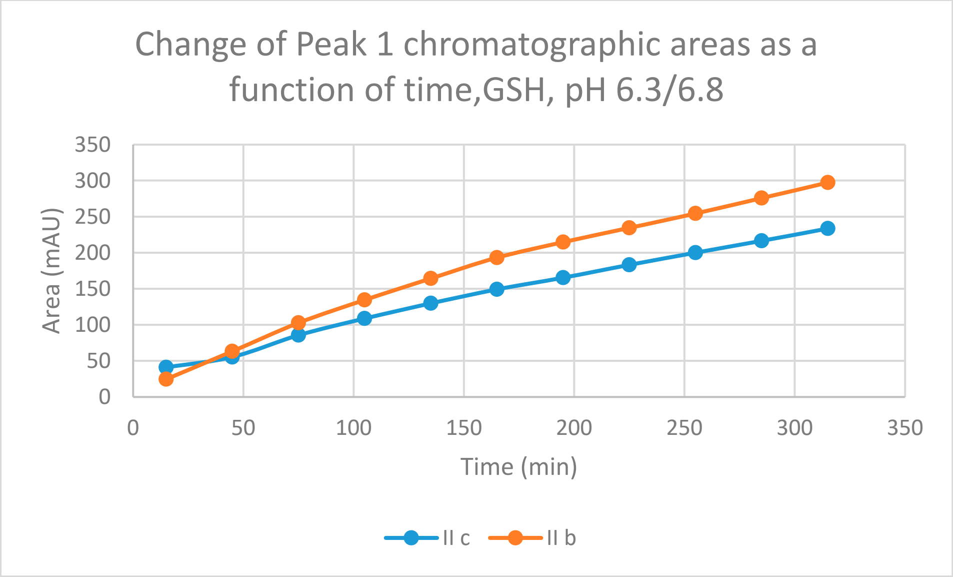

Figure S11. Change in the chromatographic peak area of adduct 1 of **II b** and **II c** in the chalcone–GSH incubations at pH 6.3/6.8.

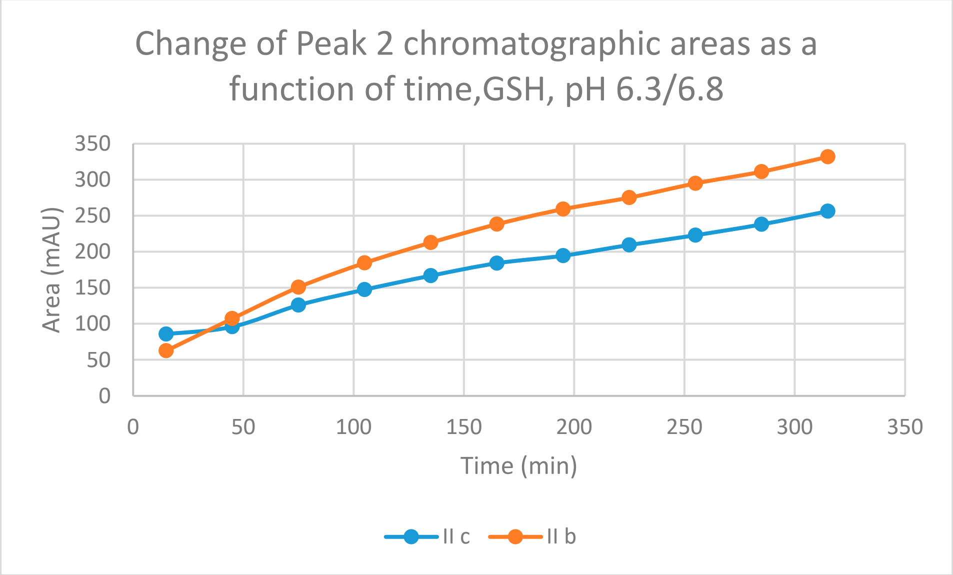

Figure S12. Change in the chromatographic peak area of adduct 2 of **IIb** and **IIc** in the chalcone–GSH incubations at pH 6.3/6.8.

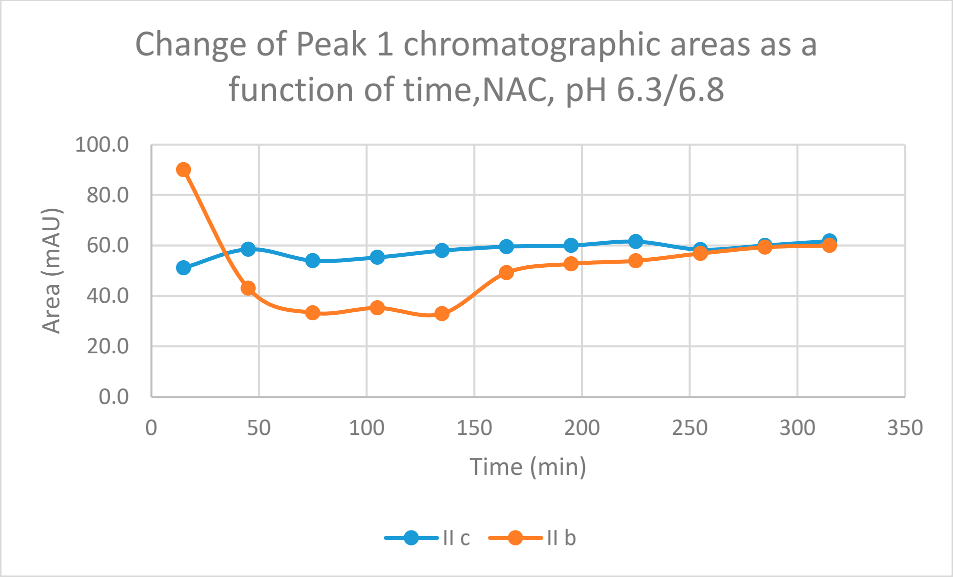

Figure S13. Change in the chromatographic peak area of adduct 1 of **IIb** and **IIc** in the chalcone–NAC incubations at pH 6.3/6.8.

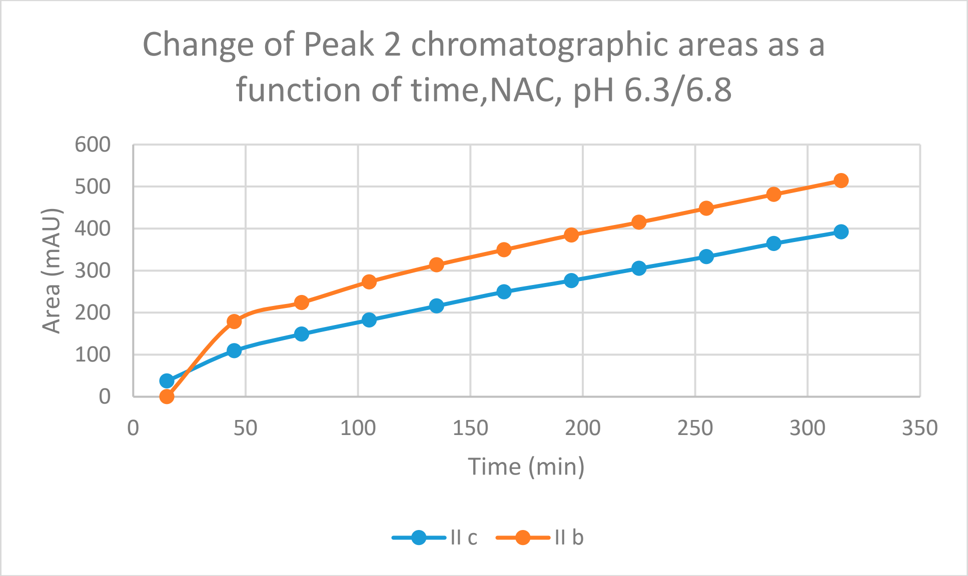

Figure S14. Change in the chromatographic peak area of adduct 2 of **IIb and IIc** in the chalcone–NAC incubations at pH 6.3/6.8.

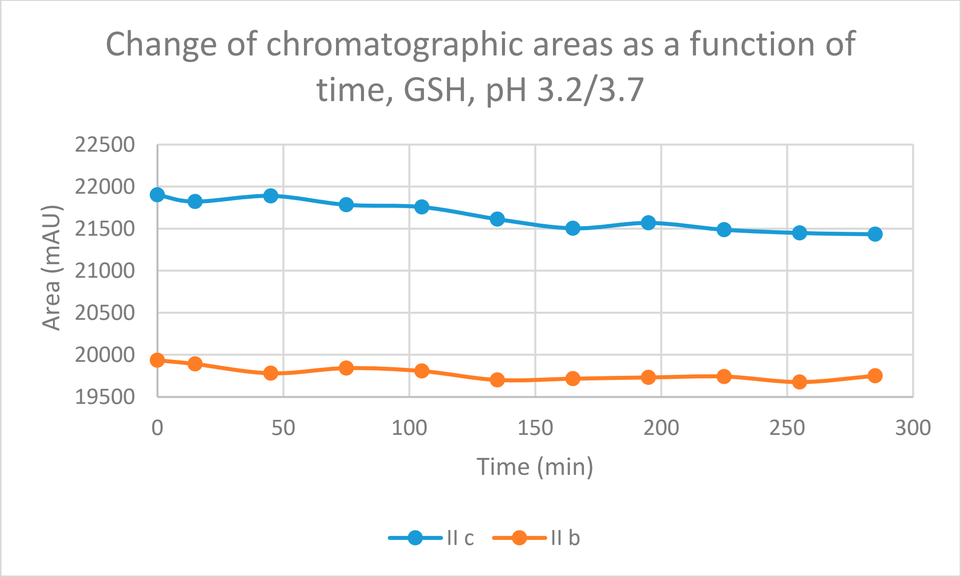

Figure S15. Change in the chromatographic peak area of **IIb and IIc** in the chalcone–GSH incubations at pH 3.2/3.7.

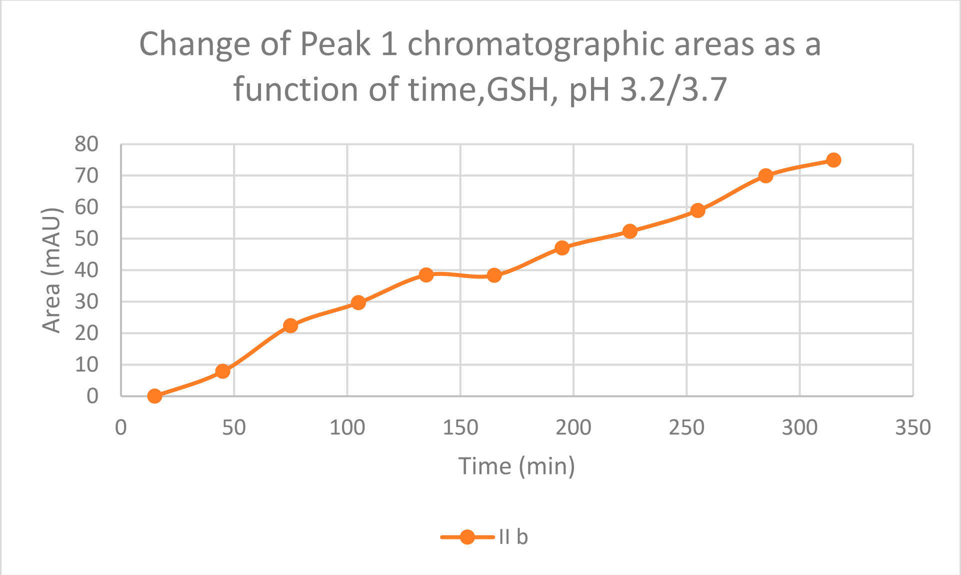

Figure S16. Change in the chromatographic peak area of adduct 1 of **IIb** in the chalcone–GSH incubations at pH 3.2/3.7.

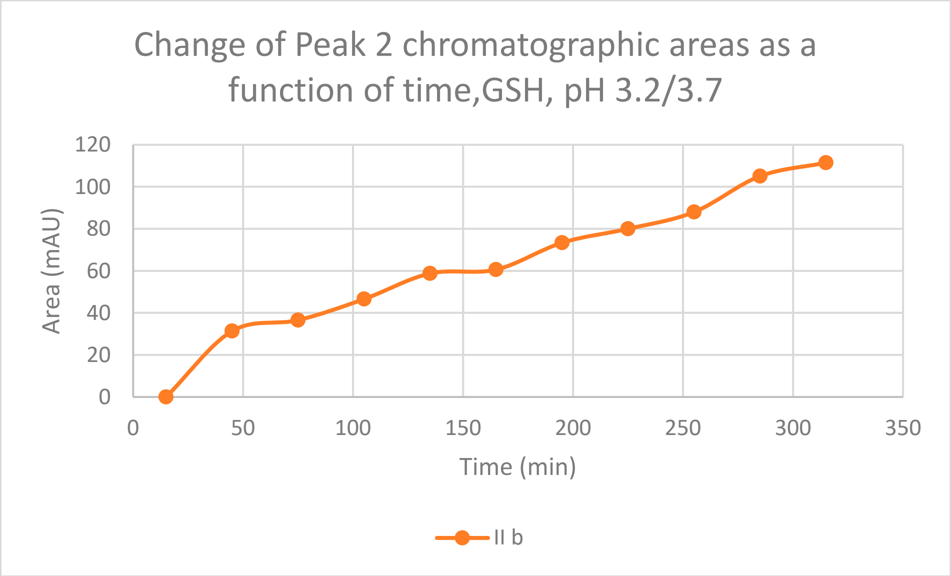

Figure S17. Change in the chromatographic peak area of adduct 2 of **IIb** in the chalcone–GSH incubations at pH 3.2/3.7.

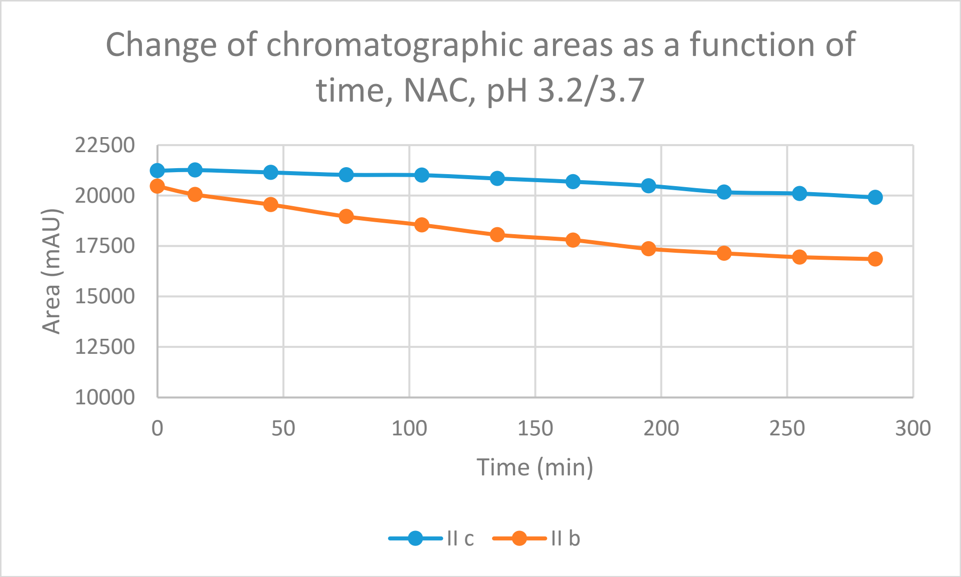

Figure S18. Change in the chromatographic peak area of **IIb and IIc** in the chalcone–NAC incubations at pH 3.2/3.7.

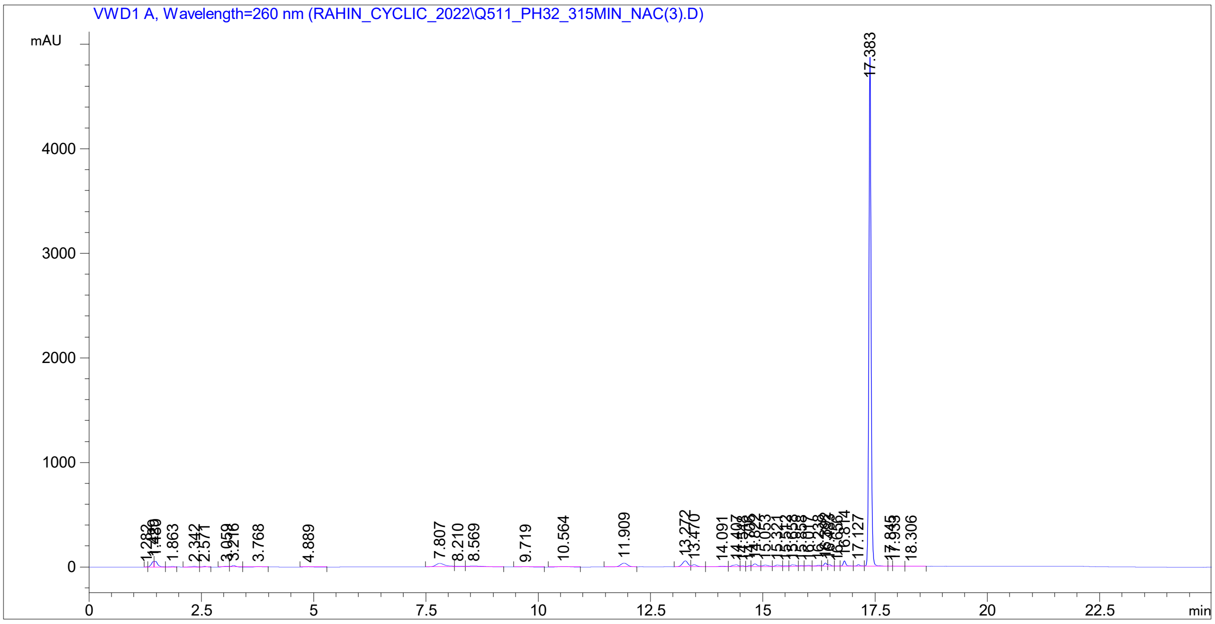

Figure S19. HPLC-UV chromatogram of pH 3.2/3.7 incubation (315 min time point) of **IIb** and NAC.

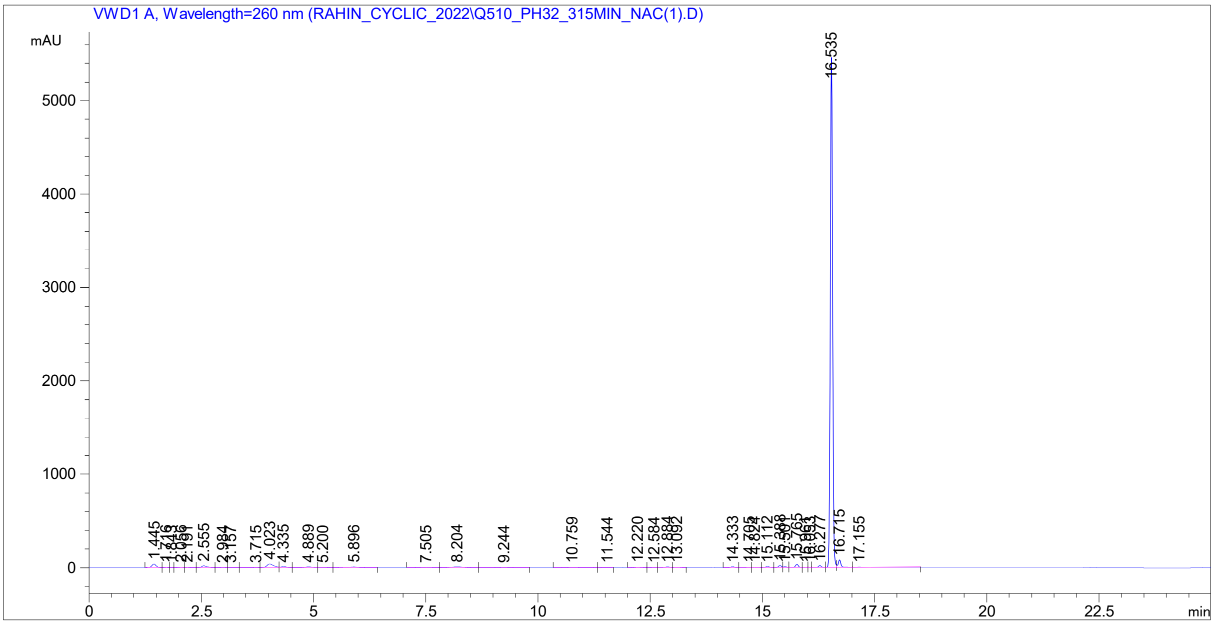

Figure S20. HPLC-UV chromatogram of pH 3.2/3.7 incubation (315 min time point) of **IIc** and NAC.

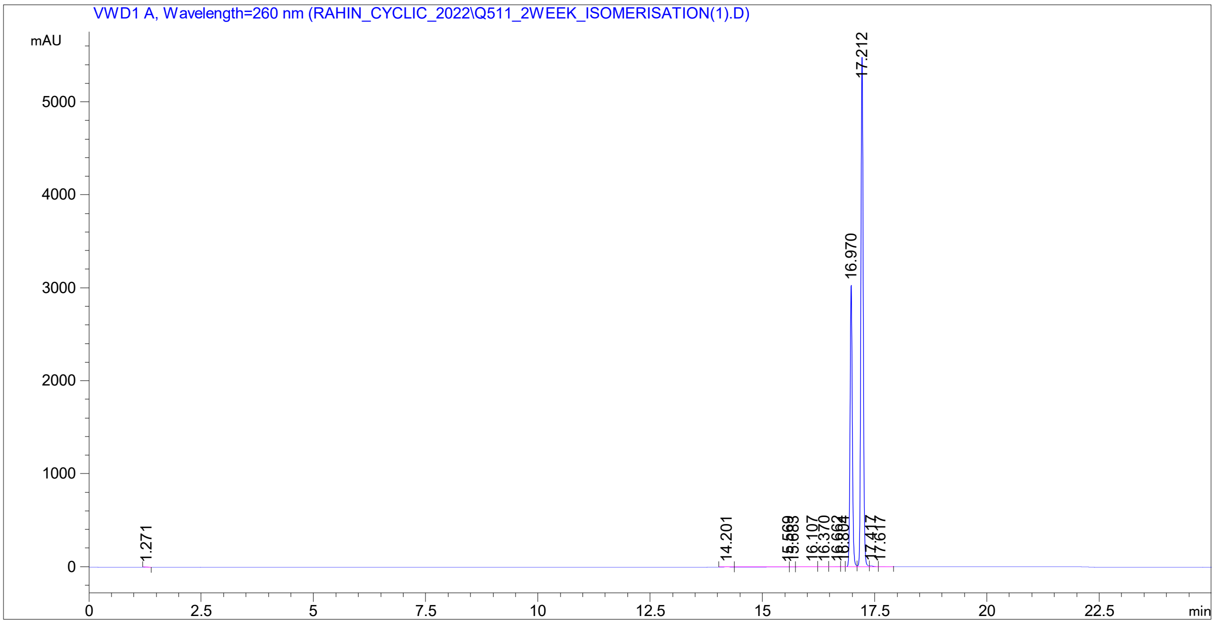

Figure S21. HPLC-UV spectrum of (*E*)(t_R 17.21 min)/(*Z*)(t_r 16.97 min) isomeric mixture of **IIb**.

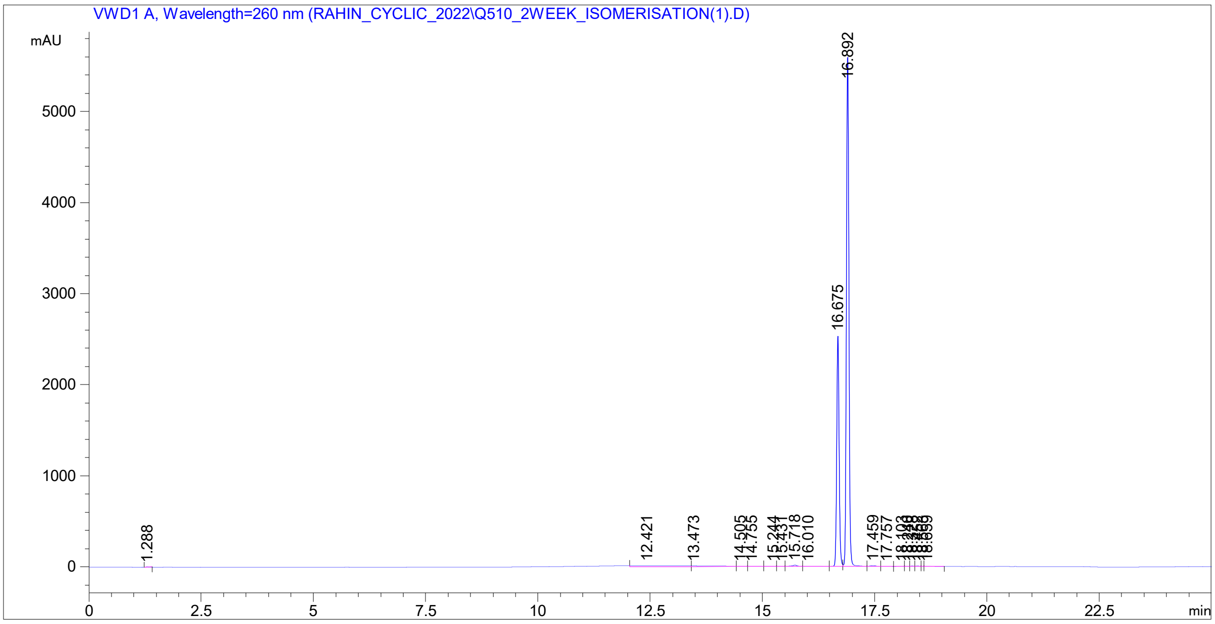

Figure S22. HPLC-UV-VIS spectrum of (*E*)(t_R 16.89 min)/(*Z*)(t_r 16.675 min) isomeric mixture of **IIc**.
